# SETD7 Promotes Cell Proliferation and Migration via Methylation-mediated TAF7 in Clear Cell Renal Cell Carcinoma

**DOI:** 10.7150/ijbs.93201

**Published:** 2024-05-19

**Authors:** Jinyuan Zhang, Baojun Duan, Fang Li, Xintao Jing, Rufeng Li, Shuang Cai, Li Cao, Qiuyu Jiang, Jing Zhou, Jiancheng Zhou, Yannan Qin, Xiaofei Wang, Dongdong Tong, Chen Huang

**Affiliations:** 1 Institute of Genetics and Development Biology, Translational Medcine Institute, Xi'an Jiaotong University, Xi'an 710301, China.; 2 Department of Medical Oncology of Shaanxi Provincial People's Hospital, Xi'an 710068, China.; 3 Department of Urology of Shaanxi Provincial People's Hospital, Xi'an 710068, China.

**Keywords:** SETD7, oncogene, lysine methylation, TAF7, clear cell renal cell carcinoma

## Abstract

SET domain containing 7(SETD7), a member of histone methyltransferases, is abnormally expressed in multiple tumor types. However, the biological function and underlying molecular mechanism of SETD7 in clear cell renal cell carcinoma (ccRCC) remain unclear. Here, we explored the biological effects of SETD7-TAF7-CCNA2 axis on proliferation and metastasis in ccRCC. We identified both SETD7 and TAF7 were up-regulated and significantly promoted the proliferation and migration of ccRCC cells. Concurrently, there was a significant positive correlation between the expression of SETD7 and TAF7, and the two were colocalized in the nucleus. Mechanistically, SETD7 methylates TAF7 at K5 and K300 sites, resulting in the deubiquitination and stabilization of TAF7. Furthermore, re-expression of TAF7 could partially restore SETD7 knockdown inhibited ccRCC cells proliferation and migration. In addition, TAF7 transcriptionally activated to drive the expression of cyclin A2 (CCNA2). And more importantly, the methylation of TAF7 at K5 and K300 sites exhibited higher transcriptional activity of CCNA2, which promotes formation and progression of ccRCC. Our findings reveal a unique mechanism that SETD7 mediated TAF7 methylation in regulating transcriptional activation of CCNA2 in ccRCC progression and provide a basis for developing effective therapeutic strategies by targeting members of SETD7-TAF7-CCNA2 axis.

## Introduction

Renal cell carcinoma (RCC), also known as renal adenocarcinoma, referred to as kidney cancer, is a tumor with a high degree of malignancy in the urinary system and one of the most common tumors, taking up approximately 80% of all renal malignancies and 3% of all cancers 1. According to the latest statistics, the incidence of kidney cancer accounts for about 5% of all male malignant tumors, ranking sixth; it also accounts for about 3% of all female malignant tumors, ranking ninth 2. Statistics show that in China, the incidence of RCC is 3.99/100,000, and the mortality rate is 1.39/100,000 3. Based on histomorphology, clinical manifestations and other characteristics, RCC is divided into three major subtypes with ≥5% incidence: clear cell renal cell carcinoma (ccRCC), papillary renal cell carcinoma (pRCC), and chromophobe renal cell carcinoma (chRCC) 4. Among them, ccRCC is the most common, representing about 75%, and its metastasis often occurs in the late stage, and the malignant degree and clinical prognosis are the worst, thus making up the majority of kidney cancer deaths 5. Since RCC is not sensitive to either chemotherapy or radiation, surgery is usually the preferred and most promising treatment, but even so, 20% to 30% of patients will develop recurrence, metastasis, or neogenesis after surgery 6, 7. Therefore, it is imperative to investigate the molecular mechanisms underlying RCC carcinogenesis and progression, which is vital for the development of potential new biomarkers and novel therapeutic strategies.

Histone methylation is an important post-translational modification catalyzed by histone methyltransferases (HMTs), which occurs on two amino acid residues, arginine and lysine, respectively 8. The methylation of histone lysine is closely related to the occurrence and development of a variety of diseases, and abnormal histone lysine methylation can lead to dysregulation of the expression of tumor suppressor genes and tumor-promoting genes, thereby playing a vital role in life activities 9. SET domain containing 7 (SETD7/SET7/KMT7), a member of the family containing SET domain proteins, was originally found to specifically monomethylate H3 Lys4, thereby regulating the expression of genes on chromatin 10. SETD7 has been identified to be a key oncogene in cancer development 11-13. Our previous study found that SETD7 promotes HCT116 and RKO cell proliferation and inhibits apoptosis in human colorectal cancer (CRC), which can serve as a potential diagnostic and prognostic biomarker for CRC 14. Furthermore, in addition to histone substrates, SETD7 is currently thought to be more common to catalyze non-histone proteins, including some transcription factors, tumor suppressors and membrane-associated receptors 15-17. As has been reported, the K87 site of RORα2 can be methylated by SETD7, thereby enhancing the binding affinity of RORα2 with the coactivation complex pontin/Tip60, inducing transcriptional activation of the CTNND1 promoter region of the target gene downstream of RORα2, and promoting the occurrence and development of prostate cancer 18. Since SETD7 can methylate a wide variety of non-histone substrates, and itself may have mutations, SETD7 may play very different roles in distinct types of tumors 19. Recent studies revealed that SETD7 can methylate the K873 of pRB, thereby increasing RB1-HP1 interactions, which in turn inhibits transcription 20; methylate DNMT1 at K142 and enhance proteasome-mediated DNMT1 degradation to exert cancer suppressive activity 21; methylate SUV39H1 at K105/123 to inhibit SUV39H1 methyltransferase activity in response to DNA damage, thereby inducing genomic instability and inhibiting cell proliferation 22. These findings suggest that understanding the mechanisms and consequences of SETD7 on methylation of cancer-relevant substrates will provide important insights into tumor progression.

TATA-box binding protein associated factor 7 (TAF7/TAFII55), as a component of the general transcription factor complex TFIID, plays a vital role in regulating transcription. TAF7 interacts with the TAF1 component and inhibits its acetyltransferase activity, which is critical for transcriptional initiation. Once the assembly of the transcriptional pre-initiation complex is complete, TAF7 is released from TAF1, allowing for transcriptional start 23. The abnormal expression of TAF7 is closely related to tumor development. Hao et al. proved that microRNA‐374c‐5p inhibits the development of breast cancer through TAF7‐mediated transcriptional regulation of DEP domain containing 1 (DEPDC1) 24. In glioblastoma, bioinformatics analysis found that TAF7 expression was upregulated, while TAF7 can promote the expression of cycle-related proteins CCNB1 and CCNA2 25. As is known, protein abundance is regulated at both transcriptional and post-transcriptional levels. A previous study had tentatively suggested that SETD7 could methylate TAF7 at K5 site 26. However, little is known about biological function and molecular mechanisms of TAF7 in ccRCC, and whether TAF7 has other methylation sites recognized by SETD7, whether the stability of TAF7 is regulated by lysine methylation and methylated-TAF7 function is far from well understood.

Our study aims to examine the role of SETD7 in mediating TAF7 protein expression and stabilization by facilitating its methylation. We reported SETD7 and TAF7 are highly expressed and exert carcinogenic functions in ccRCC. SETD7 can directly bind to TAF7 and promote its methylation at K5 and K300, thereby inhibiting its ubiquitination to maintain TAF7 stability. Moreover, methylation of TAF7 triggers an increase in downstream target CCNA2 gene activation, which in turn drives the progression of ccRCC. The study may provide useful information to help understand the underlying mechanism of methylation related to SETD7 during the development of ccRCC cancer and provide novel effective targets for cancer therapy.

## Materials and Methods

### Bioinformatics data analysis

The expression of SETD7 and TAF7 in pan-cancer tissues and normal tissues were analyzed using the data from TCGA database; String database was used to analyze SETD7 and TAF7 interaction network; GEPIA database was used to analyze SETD7 and TAF7 correlation; Protein docking analysis between SETD7 and TAF7 was performed using PyMOL Software; Volcano maps were plotted by the R package of ggplot 2; Kyoto Encyclopedia of Genes and Genomes (KEGG) pathway enrichment and Gene Set Enrichment Analysis (GSEA) were performed using the R package clusterProfiler with differentially expressed genes (DEGs); The expression of CCNA2 were analyzed using the data from TCGA database; UCSC database was used to analyze downstream target genes of TAF7 gene. The specific URL of the bioinformatics database as well as the version information of the drawing package are shown in Table S1.

### Patient samples

All clear cell renal cell carcinoma (ccRCC) tumor and adjacent normal tissue samples were collected from Shaanxi Provincial People's Hospital, Xi'an, China and used for the immunohistochemistry staining experiment. Both tumor and non-tumor tissues were histologically confirmed. The consent of each patient was obtained before the samples were collected and the study was conducted following the protocol approved by the Biomedical Ethics Committee of Shaanxi Provincial People's Hospital.

### Immunohistochemistry (IHC)

IHC staining was performed on the tissue specimens collected from ccRCC patients. The tissue samples were fixed in 4% paraformaldehyde, embedded in paraffin and cut into 5 μm thickness with a microtome. The sections were deparaffinized with 100% xylene and hydrated with graded alcohol (100, 95, 80, and 70%) and then antigen retrieval and blocking were applied. Then the sections were incubated with primary antibodies (SETD7 and TAF7) at 4 °C overnight, followed by incubation with secondary antibody at room temperature. Subsequently, the immunostaining was performed with 3,3'- diaminobenzidine (DAB, ZSGB-BIO) and finally, tissues were counterstained with hematoxylin. Images were obtained with microscopic analysis (Media Cybernetics, United States) and the percentages of staining were quantified by using ImageJ v1.8.0 (Wayne Rasband, USA).

### Cell culture

Human ccRCC cell lines 786-O and CAKI-1 as well as human tubular epithelial cell line HK-2 were purchased from the Cell Bank (Shanghai Genechem Co., Ltd., Shanghai, China). All cell lines had been authenticated by the Cell Bank. 786-O and HK-2 were cultured in RPMI-1640 supplemented with 10% (v/v) fetal bovine serum (FBS, Gibco, NY, USA) and 1% (v/v) penicillin/streptomycin (PS, solarbio, Beijing, China). CAKI-1 cells were cultured in McCoy's 5a Modified Medium supplemented with 10% (v/v) FBS and 1% (v/v) PS. All cell lines were cultured in a humidifying atmosphere with 5% CO_2_ at 37 °C and passaged when reaching approximately 90% confluency. All cells were regularly tested for mycoplasma contamination and were negative.

### siRNA synthesis, plasmid construction, and transfection

siRNAs targeting SETD7, TAF7 and CCNA2 were designed and synthesized by GenePharma (Shanghai, China). The negative control was a non-sense siRNA (NC-siRNA). The specific siRNA sequences are shown in Table S2. The plasmids of SETD7 and TAF7 (WT) were purchased from the company (Genechem, Shanghai, China). TAF7-K5R, TAF7-K300R and TAF7-2KR (K5R/K300R double mutant) plasmids were constructed by using Fast Mutagenesis System kit (TransGen Biotech) on the basis of TAF7-WT. Using jetPRIME™ reagent (Polyplus-Transfection, France) for siRNAs and plasmids transfection according to manufacturer's instructions.

### RNA extraction and quantitative real-time PCR (qRT-PCR)

Total RNA was extracted from the cell lines or frozen tissue using TRIzol® reagent (Genestar, Shanghai, China) following the manufacturer's methods. RNA concentrations were examined using Spectrophotometer (DeNovix, USA). Reverse transcription of mRNA to cDNA and quantitative real-time PCR were performed by using cDNA Synthesis Kit and SYBR Green PCR kit (Genestar, Shanghai, China) according to the manufacturer's instructions. The primers are presented in Table S2. All qRT-PCR reactions for each sample were performed in triplicate, using the IQ5 Multicolor qRT-PCR Detection System (Bio-Rad, USA). GAPDH was used as an internal control in all experiments and the 2-ΔΔCt method was used to calculate relative expression levels.

### Protein extraction and western blotting

Total proteins were extracted from the tissues and cells using RIPA buffer containing protease and phosphatase inhibitor (Beyotime, Beijing, China). The protein concentrations were determined by using the BCA Protein Assay Kit (Beyotime, Beijing, China). The same amounts of protein lysates were run on 10% SDS-PAGE gels and then transferred to methanol-activated PVDF membranes (Roche, Indianapolis, IN, USA). After transfer, the membranes were blocked with 5% nonfat milk in TBST for 1 h at room temperature. And then incubated with primary antibodies at 4 °C overnight, followed by the corresponding secondary antibody for 1 h at room temperature the next day. After completion of the incubation, protein bands were detected by chemiluminescence using ECL kit (Pierce, Rockford, IL, USA) and imaging signals were acquired by ChemiDoc™ Touch (Bio-Rad, USA). The primary and secondary antibodies used are shown in Table S3.

### MTT assay for cell viability

The ccRCC cell lines of 786-O and CAKI-1 were seeded in 96-well plates (3×10^3^ cells/well) respectively and siRNAs or plasmids were used for transfection the next day. After continuous culture for 24 h, 48 h, and 72 h, 10 μL MTT (5 mg/ml) was added to each well and then incubated at 37 °C for 4 h. Then discarded the supernatant and added 150 μl dimethyl sulfoxide (DMSO) to dissolve the purple crystals. Finally, the cell activity was determined at 492 nm absorbance using a microplate reader (FLUOstar Omega, BMG, Germany).

### Colony forming assay

786-O and CAKI-1 cells transfected after 24 h were trypsinized and planted in 12-well plates (5×10^2^ cells/well). Cells were cultured for 1-2 weeks, and when the single cell colony grew to the size of rice grain, the colonies were then fixed with 4% paraformaldehyde for 15 min at room temperature and stained with 0.1% crystal violet for 30 min. After washing with PBS, the colony images were acquired and numbers were analyzed using Quantity One® software (Bio-Rad, USA).

### Flow cytometry analysis of cell cycle/apoptosis

For flow cytometry analysis of cell cycle: 786-O and CAKI-1 cells were harvested by trypsinization 24 h after transfection, washed twice with PBS, and fixed with ice-cold ethanol (70%) at 4 °C overnight. After washing twice again, the cells were then suspended in 150 μl RNase A (100 μg/ml) and 150 μl propidium iodide (50 μg/ml) for 15 min at room temperature. The distribution of cell cycle stages was examined by flow cytometry (FACSCalibur, BD, Biosciences, USA).

For flow cytometry analysis of cell apoptosis: 786-O and CAKI-1 cells were harvested by trypsinization 48 h after transfection, washed twice with PBS, the cells were resuspended in 1× binding buffer, and stained using Annexin-V-FITC/PI apoptosis detection kit (Invitrogen, Carlsbad, California, USA) according to the manufacturer's methods. The level of cell apoptosis was analyzed using flow cytometry (FACSCalibur, BD, Biosciences, USA).

### Wound-healing, transwell migration Assay

For wound-healing assay, 786-O and CAKI-1 cells were seeded into 6-well plates and transfected. The cells were scratched with a 200 μl disposable gun head to make a wound when the cell density reached 80-90%. Subsequently, the detached cells were removed with PBS, 1% FBS fresh medium was added in the wells. The wound closures were observed and photographed under a microscope (Nikon, Japan) at 0 h, 12 h, 24 h for 786-O and at 0 h, 24 h, 48 h for CAKI-1, respectively.

Cell migration assays were performed using transwell chambers (8 μm pore size; Millipore, Billerica, MA, USA) and inserted into a 24-well plate. Differently treated cells were plated in the upper chambers at a density of 3 × 10^4^ cells in 200 μl of serum-free medium, and 600 μl of complete medium was added to the lower chambers. Continue to incubate the 786-O for 24 h and CAKI-1 for 48 h, cells that did not migrate via the pores were removed by a cotton swab. Cells adhered to the bottom surface of each membrane were fixed with 4% paraformaldehyde for 15 min, and stained with 0.1% crystal violet for 30 min. After acquiring images using a microscope (Nikon, Japan) with a magnification of 10 ×, the migrated cells were dissolved in 33% glacial acetic acid and optical density (OD) was measured using a microplate reader (FLUOstar Omega, BMG, Germany) at 570 nm.

### Immunofluorescence microscopy

After 48 hours, the transfected ccRCC cells were fixed with 4% paraformaldehyde for 15 min, washed three times with PBS, followed by permeabilization with 0.1% Triton X-100. And then cells were blocked with 10% nonfat milk in PBS for 1 h at room temperature and incubated with primary antibodies (SETD7, TAF7, Flag and His) overnight at 4 °C. Subsequently, the cells were stained using appropriate fluorochrome-conjugated secondary antibody for 1 h in the dark the next day. DAPI was added to counterstain cells for 10 min after washing with PBS. A fluorescence microscope (Leica, TCS SP8 DIVE, Germany) was used to measure immunofluorescent signals.

### Co-immunoprecipitation assays

Cells were harvested and washed three times in cold PBS, and then lysed in lysis buffer (150 mM NaCl, 1% NP-40, 50 mM Tris-HCl, pH8.0, protease inhibitor) to extract total proteins. After incubating on ice, the lysates were centrifuged at 12,000 g for 10 min at 4 °C. Then supernatant was collected and incubated with primary antibodies (SETD7, TAF7, Flag or His) overnight at 4 °C with gentle shaking, followed by adding Dynal magnetic beads (Invitrogen, CA, USA) for 2-4 h the next day. The beads were washed three times with PBS and resuspend in 2 × loading buffer. Then beads were boiled at 100 °C for 10 min. The beads were thrown away and the liquid was collected for immunoblotting analysis.

### Luciferase reporter assay

The CCNA2 promoter region containing TAF7-binding site was cloned into pGL3 luciferase vector, between Kpnl and XhoI sites. The constructed wild-type (WT) or mutant (MUT) TAF7/CCNA2 vectors were co-transfected with the indicated plasmids into HEK293 cells, respectively. After 48 h, luciferase activity assay was measured using Dual Luciferase Assay System (Promega) according to the manufacturer's instructions with FLUOstar OPTIMA (BMG). Renilla luciferase activity was used as an internal standard.

### Tumorigenicity assay, tumor metastasis assay in nude mice

A total of 24 male, 5-week-old BALB/C nude mice were used to examine tumorigenicity and tumor metastasis. All mice were purchased from the Experimental Animal Center of Xi'an Jiaotong University and maintained in accordance with the guidelines of the Animal Care and Use Committee of Xi'an Jiaotong University. The animals were observed for 1 week prior to the start of the experiments.

For tumorigenicity assay, we generated CAKI-1 cells that stably silenced SETD7 in the first experiment. 1 × 10^6^ LV-Control group and LV-shSETD7 group cells suspended in 0.1 ml PBS respectively were injected into subcutaneously both sides of the mice's groin to eliminate individual differences of animals (n = 4/group). In the second experiment, 12 mice were randomly divided into three groups (shSETD7+TAF7-WT, shSETD7+TAF7-K5R and shSETD7+TAF7-K300R). 1 × 10^6^ CAKI-1 cells suspended in 0.1 ml PBS were subcutaneously injected into the right femoral area of each mouse. For both experiments, tumor growth was observed every 4 days. At the 31th day after injection, the nude mice were anesthetized by inhalation of 3% isoflurane and given a subcutaneous dose of carprofen (8 mg/kg) for euthanasia. Then xenograft tumors were excised, weighed and photographed. Tumor size was measured using vernier calipers and tumor volume was calculated according to the formula: volume = length × (width^2^)/2. The tumor tissues were frozen for qRT-PCR and western blotting.

For tumor metastasis assay, as mentioned before, we generated CAKI-1 cells stably silencing SETD7 using lentiviral vectors. LV-Control group and LV-shSETD7 group (1 × 10^6^ cells suspended with 0.1 ml of PBS) were injected into mice by tail vein (n = 4/group). 21 days after injection, the mice were anesthetized by isoflurane/oxygen and bioluminescence images were acquired and analyzed using IVIS Spectrum (Xenogen Corp., Alameda, CA, USA). After the mice were sacrificed with euthanasia, the organs were resected and images of metastasis were taken with IVIS Spectrum.

### Statistical analysis

All experiments were repeated at least in triplicate and statistical analysis was carried out using SPSS Statistics 18.0 (Chicago, IL, USA). Data were presented as the mean ± standard error of the mean (SEM) and Student's t-test (two-tailed) was used for comparisons between two independent groups. One-way ANOVA followed by multiple comparisons using Dunnett's test was performed to analyze differences among more than two groups. Pearson correlation analysis was conducted to estimate the association of SETD7 with TAF7, as well as TAF7 with CCNA2. *P* < 0.05 was considered statistically significant.

## Results

### SETD7 is up-regulated in ccRCC and promotes the proliferation and migration of ccRCC cells *in vitro*

**Figure 1:**
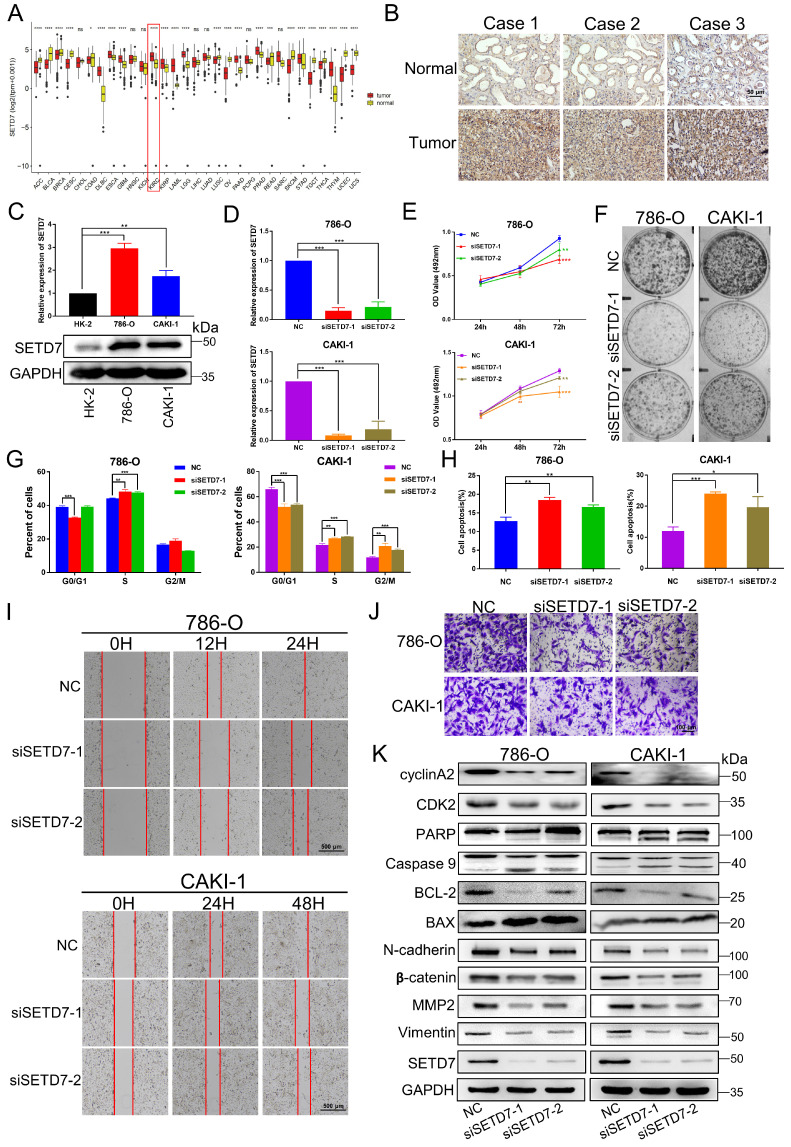
** SETD7 is upregulated in ccRCC and silencing of SETD7 inhibits the biological function of ccRCC cells. A,** The expression level of SETD7 in 31 Pan-cancer which analyzed by TCGA databases. **B,** Representative immunohistochemical images of SETD7 in human renal clear cell carcinoma tissues and adjacent normal tissues. **C,** The mRNA and protein expressions of SETD7 were detected in ccRCC cell lines (786-O, CAKI-1) and normal tubular epithelial cell line (HK-2), analyzed by qRT-PCR and western blotting. **D,** The mRNA expression of SETD7 were detected to confirm the knockdown efficiency of two siRNAs in 786-O and CAKI-1 cells, analyzed by qRT-PCR. **E,** MTT assay was used to detect the effect of silencing SETD7 on the proliferation of 786-0 and CAKI-1 cells. **F,** Colony formation assays was used to detect the effect of silencing SETD7 on the clonogenic ability of 786-0 and CAKI-1 cells. **G,** Flow cytometry was performed to detect the effects of silencing SETD7 on cell cycle progression in 786-O and CAKI-1 cells, and the percentages of G1, S, G2 phase of cell cycle were calculated. **H,** Flow cytometry was performed to detect the effects of silencing SETD7 on cell apoptosis in 786-O and CAKI-1 cells, and the percentage of cell apoptosis was calculated. **I,** The effects of silencing SETD7 on 786-O and CAKI-1 cells migration were determined by wound-healing assay and transwell assay (**J**). **K,** Expression changes of cell cycle, apoptosis and migration-related molecules by silencing SETD7 in 786-O and CAKI-1 cells were detected by western blotting. *p < 0.05, **p < 0.01, ***p < 0.001.

Based on The Cancer Genome Atlas (TCGA) datasets of pan-cancer, there are as many as 11 types of cancers with high SETD7 expression, which are diffuse large B cell lymphoma (DLBC), Esophageal carcinoma (ESCA), Glioblastoma multiforme (GBM), Kidney renal clear cell carcinoma (KIRC), Kidney renal papillary cell carcinoma (KIRP), Acute Myeloid Leukemia (LAML), Brain Lower Grade Glioma (LGG), Pancreatic adenocarcinoma (PAAD), Prostate adenocarcinoma (PRAD), Stomach adenocarcinoma (STAD) and Thymoma(THYM) (Fig. 1A). Next, we performed IHC staining. The results showed that the expression of SETD7 in ccRCC tissues was significantly higher than that in adjacent normal tissues (Fig. 1B; Fig. S1A). Furthermore, we compared the expression of SETD7 at mRNA and protein levels in two ccRCC cell lines (786-O and CAKI-1) by qRT-PCR and western blotting, and took the normal renal epithelial cell line HK-2 as the control. Compared with HK-2, the expressions of SETD7 mRNA and protein were up-regulated in both two ccRCC cell lines (Fig. 1C). Then in order to examine the role of SETD7 in ccRCC cell progression, we designed and synthesized two small interfering RNAs (siRNAs) that silenced the expression of SETD7, qRT-PCR and western blotting were performed to detect the knockdown efficiency of siRNAs and as shown in Fig. 1D and Fig. S1B, the mRNA and protein expression of SETD7 was successfully inhibited. MTT assay showed that knockdown of SETD7 in 786-O and CAKI-1 cells significantly inhibited cell proliferation (Fig. 1E). Consistent results were also observed in the colony formation assay. As shown in Fig. 1F and Fig. S1C, SETD7 silencing induced a more distinctive decrease of cell colony in 786-O and CAKI-1 cells. Next, we investigated the effect of SETD7 on cell cycle and apoptosis, flow cytometry results indicated that SETD7 knockdown led to a greater decrease in G1 phase cells and a greater increase in S phase cells (Fig. 1G; Fig. S1D). To confirm this finding, we also showed that SETD7 siRNA significantly promoted cell apoptosis in 786-O and CAKI-1 cells (Fig. 1H; Fig. S1E). Meanwhile, to explore the effect of SETD7 on the migration ability of ccRCC cells, wound-healing and transwell assays were performed. The results showed the wound healing rate of cells after silencing SETD7 was significantly slower than that of the control group (Fig. 1I; Fig. S1F). Consistent with these results, transwell assays also revealed an inhibitory effect of silencing SETD7 on cell migration capacity of ccRCC cells (Fig. 1J; Fig. S1G). Subsequently, western blotting analyses were also performed to examine the potential molecular mechanisms by which SETD7 modulates ccRCC progression. As shown in Fig. 1K, silencing SETD7 suppressed the expression of S phase-related proteins CDK2 and cyclinA2 indicating S-phase arrest. Besides, knockdown SETD7 promoted cell apoptosis by dynamically regulating the activation of pro-apoptotic protein Bax and the inactivation of anti-apoptotic protein Bcl-2, accompanied by increased levels of cleaved-PARP and cleaved-Caspase 9. In addition, SETD7 was positively correlated with migration-promoting protein N-cadherin, β-catenin, MMP2 and vimentin levels. Taken together, the results of this section indicated that SETD7 is overexpressed in ccRCC and acts as an oncogene promoting ccRCC progression *in vitro*.

### SETD7 promotes ccRCC tumor growth and metastasis *in vivo*

**Figure 2:**
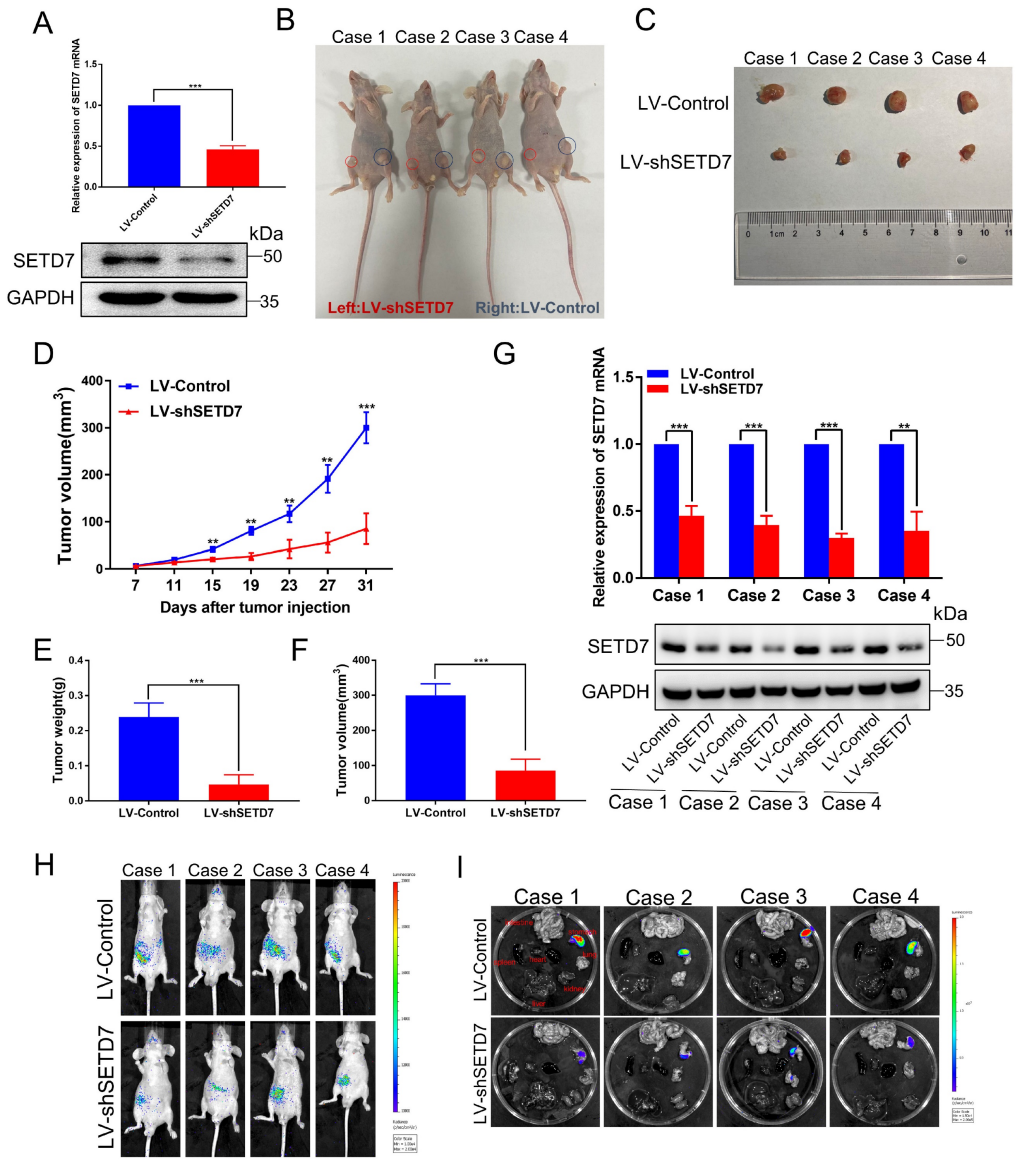
** Silencing of SETD7 inhibits tumor growth and metastasis *in vivo*. A,** The mRNA and protein expression of SETD7 in LV-shSETD7 cells was detected by qRT-PCR and western blotting. **B,** Gross morphology of tumors injected with either LV-Control or LV-shSETD7 cells after 31 days (n = 4). **C,** Morphology of excised tumors from nude mice. **D,** Growth curves of tumor volume were generated every 4 d for 31 d. **E,** Weight statistics of excised tumors. **F,** Volume statistics of excised tumors. **G,** The mRNA and protein expression levels of SETD7 were analyzed by qRT-PCR and western blotting in removed tumors. **H,** Image analysis was performed on nude mice to assess tumor metastasis on the 21th day after injection. **I,** Organs were resected and images of metastasis were shown. *p < 0.05, **p < 0.01, ***p < 0.001.

To confirm these results *in vivo*, we successfully constructed a stable CAKI-1 cell line that knocked down SETD7 and then performed tumorigenesis and tumor metastasis assay in nude mice. As shown in Fig. 2A, qRT-PCR and western blotting showed that both mRNA and protein of SETD7 were significantly downregulated in LV-shSETD7 cells compared to LV-Control cells. Then we subcutaneously injected LV-shSETD7 as well as LV-Control cells into the groin on both sides of BALB/C nude mice to observe tumor growth. At the 31th day after injection, the tumor formed on the side of LV-shSETD7 group in the same nude mouse was significantly smaller than LV-Control group. (Fig. 2B, C). The tumor growth curve was plotted using tumor volume measured during tumor formation, and the results indicated that tumors in LV-shSETD7 infection group grew slower than in LV-Control group (Fig. 2D). The weight and volume of the excised solid tumor were then measured. The results showed that the tumor weight and volume of LV-shSETD7 group were significantly lighter and smaller compared to LV-Control group (Fig. 2E, F). Expression of SETD7 at the RNA and protein levels from LV-shSETD7 and LV-Control solid tumors were detected by qRT-PCR and western blotting. The results suggested that SETD7 in the LV-shSETD7 group was significantly down-regulated compared with the LV-control group (Fig. 2G). In addition, to further evaluate the role of SETD7 in ccRCC metastasis *in vivo*, we established a tail vein metastasis model by injecting CAKI-1 cells into nude mice and detected the distribution and size of the tumor on the 21th day after injection. The results showed that LV-shSETD7 group significantly suppressed ccRCC cells metastasis in contrast with the LV-Control group (Fig. 2H, I; Fig. S1H, I). Overall, these results suggested that SETD7 directly promotes the growth and metastasis of ccRCC tumors *in vivo*.

### SETD7 and TAF7 could bind to each other

**Figure 3:**
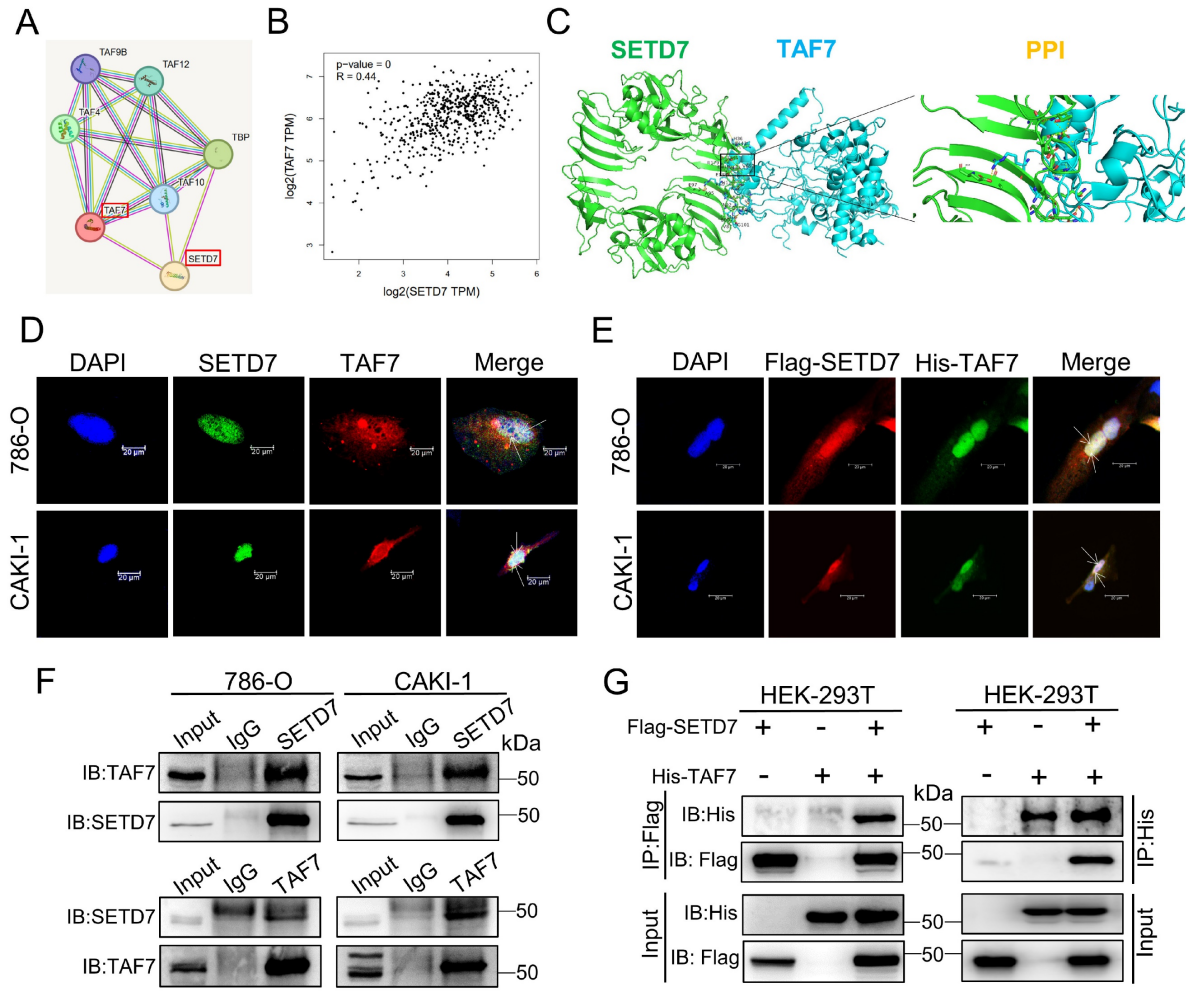
** Binding and co-localization between SETD7 and TAF7. A,** The interaction between SETD7 and TAF7 was analyzed by STRING database. **B,** The correlation between the expression of SETD7 and TAF7 in ccRCC was analyzed by GEPIA database. **C,** Representative images of protein docking analysis. SETD7 is shown in green, and TAF7 is shown in blue. Protein-protein interaction positions marked by yellow lines. **D,** Representative images of immunofluorescence staining of DAPI, SETD7 and TAF7 in 786-O and CAKI-1 cells showed co-localization between SETD7 and TAF7 in the nucleus. **E,** Representative images of immunofluorescence staining of DAPI, Flag-SETD7 and His-TAF7 in 786-O and CAKI-1 cells. Results indicated co-localization between Flag and His in the nucleus. **F,** Co-immunoprecipitation (Co-IP) assays were used to detect interaction of endogenous SETD7 and TAF7 in 786-O and CAKI-1 cells. **G,** HEK-293T cells were co-transfected with Flag-SETD7 and His-TAF7 expression vectors. Co-IP assays were performed to detect protein interactions between exogenous SETD7 and TAF7.

To further investigate the relationship between SETD7 and TAF7, we performed immunofluorescence (IF) staining and Co-immunoprecipitation (Co-IP) assays. First of all, STRING database analysis revealed possible interactions between SETD7 and TAF7 (Fig. 3A), and GEPIA database analysis showed that there was a significant positive correlation between the expression of SETD7 and TAF7 in ccRCC (Fig. 3B). In addition, protein docking analysis was used to predict the binding between SETD7 and TAF7, and the results hinted that the two proteins bound to each other (Fig. 3C). Moreover, IF staining was used to examine the location of SETD7 and TAF7 binding in ccRCC cells. As shown in Fig. 3D, SETD7 and TAF7 were co-localized in the nucleus. Consistent with these results, Flag-SETD7 and His-TAF7 were also significantly co-localized in the nucleus when SETD7 and TAF7 were overexpressed (Fig. 3E). Co-IP assays results confirmed the direct protein interaction of endogenous SETD7 and TAF7 in 786-O and CAKI-1 cells (Fig. 3F). Besides, additional Co-IP assays were performed using Flag-SETD7 and His-TAF7. As shown in Fig. 3G, ectopically expressed Flag-SETD7 interacted directly with His-TAF7, confirming their exogenous binding. These results demonstrated that there is a binding and colocalization relationship between SETD7 and TAF7.

### SETD7 methylates TAF7 at lysine 5 and 300

**Figure 4:**
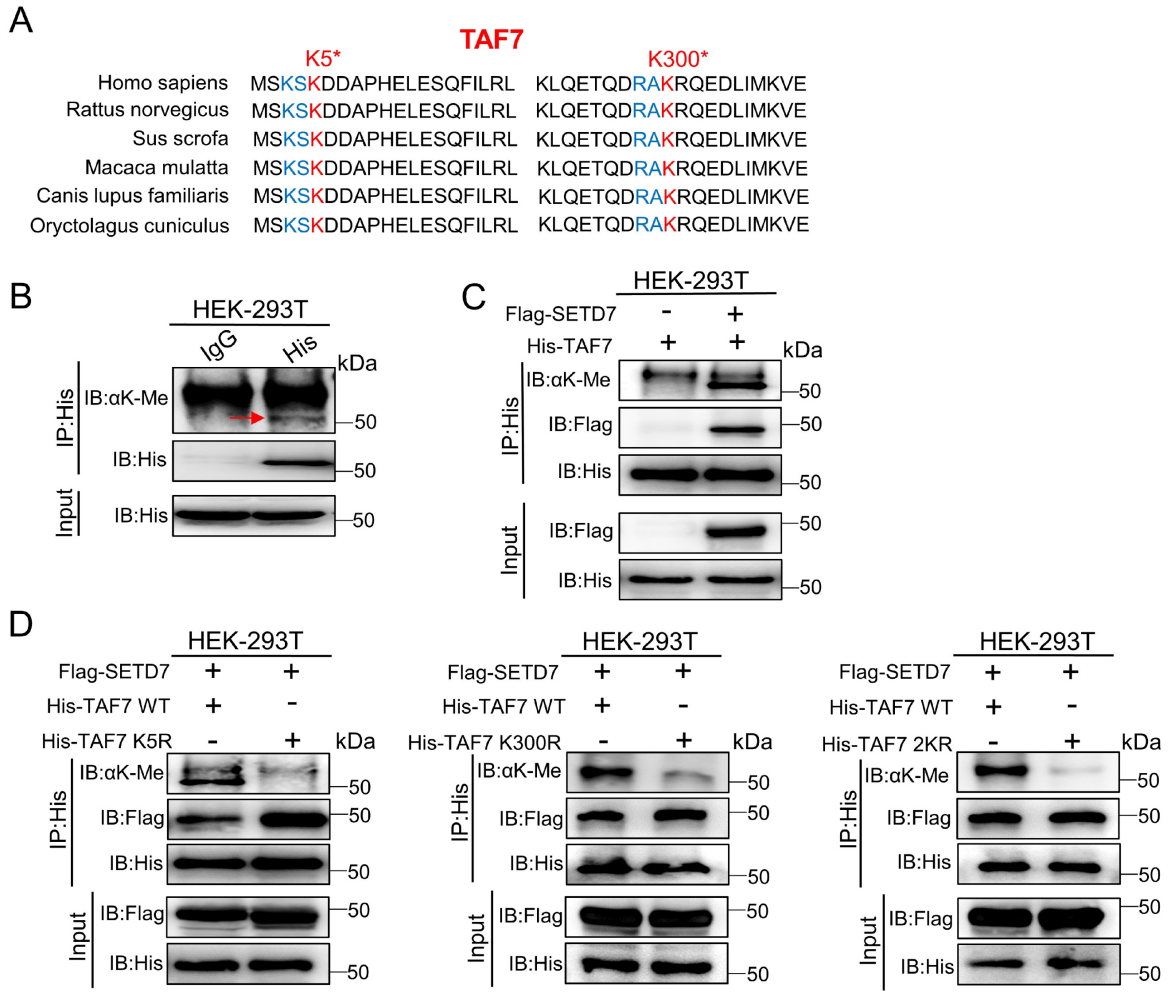
** SETD7 methylates TAF7 at lysine K5 and K300. A,** Sequence alignment of TAF7 domain containing the SETD7 recognized [K/R]-[A/S/T]-K-X motif from diverse species. TAF7 residues at K5 and K300 were denoted in the protein sequences. The positions of the K5 and K300 were highlighted in red, and nearby identifiable lysine sites were highlighted in blue. **B,** Pan-lysine methylation of TAF7 in HEK-293T cells was demonstrated using Co-IP. **C,** Co-IP showed the pan-lysine methylation status of TAF7 after overexpressing SETD7 in HEK-293T cells. **D,** HEK-293T cells were co-transfected with Flag-SETD7 and different His-TAF7 WT or K5R, K300R, 2KR mutant vectors, respectively. Cell lysates were immunoprecipitated with anti-His antibody, followed by immunoblotting analysis by anti-lysine (K) methyl antibody to detect pan-lysine methylation status of TAF7.

As a histone lysine methyltransferase, SETD7 promotes the addition of methyl to its histone substrate. Furthermore, it has recently been reported that SETD7 interacts with non-histone proteins and promotes non-histone proteins methylation. Additionally, since our data showed that SETD7 interacts directly with TAF7 (Fig. 3), we speculated that TAF7 may be a catalytic substrate of SETD7. Next, to corroborate this, a series of IP assays were conducted in HEK-293T cells. We discovered that TAF7 contains an evolutionary conserved histone H3K4-like SETD7 recognition motif ([K/R]-[A/S/T]-K-X) among the mammals analyzed, thus inferring that the lysine 5 (K5) and lysine 300 (K300) sites of TAF7 as “hit target” for methylation (Fig. 4A). Subsequently, we tested the methylation status of TAF7. Western blotting analyses showed that the presence of methylated lysines on TAF7 in HEK-293T cells (Fig. 4B). Next, an IP assay was performed in SETD7 overexpressed cells, and the results revealed that the methylation status of TAF7 was significantly elevated compared to control cells (Fig. 4C). These data displayed that SETD7 is crucial for TAF7 lysine methylation. To assess the essentiality, we used site-directed mutagenesis to generate three His-tagged TAF7 mutants named K5R, K300R and 2KR (K5R/K300R double mutant) in which one or both lysine residues were replaced by arginine to eliminate lysine methylation. Then we transfected TAF7-WT, K5R, K300R and 2KR plasmids in HEK-293T cells and detected total methylation levels of TAF7 proteins. Co-IP assays results showed that K5R, K300R and 2KR mutations significantly weakened or eliminated overall TAF7 methylation levels compared to TAF7-WT, suggesting that K5 and K300 are the major lysine methylation sites of TAF7 (Fig. 4D).

### TAF7 K5 and K300 methylation promote the stabilization of TAF7

**Figure 5:**
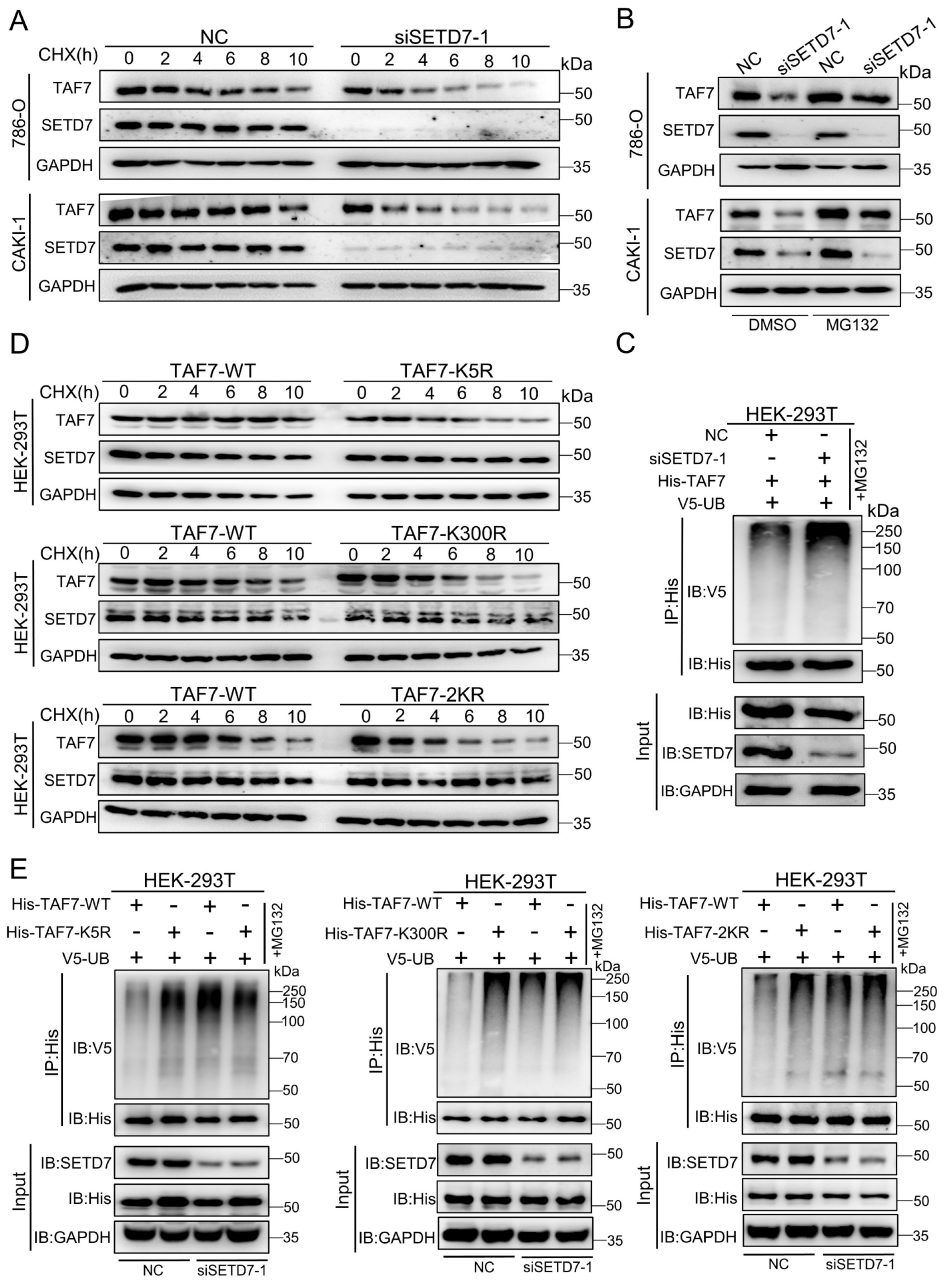
** TAF7 K5 and K300 methylation promote the stabilization of TAF7. A,** Western blotting analyses indicated protein levels of SETD7 and TAF7 in NC and SETD7 knockdown 786-O and CAKI-1 cells treated with CHX for the indicated time. **B,** 786-O and CAKI-1 cells were transfected with NC or SETD7 siRNA-1 and then treated with MG132 or DMSO for 10 h. Western blotting analyses were performed to assess protein levels of SETD7 and TAF7. **C,** HEK-293T cells were co-transfected with His-TAF7+NC or His-TAF7+siSETD7-1 and then treated with MG132 for 10 h. Cell lysates were immunoprecipitated by anti-His antibody, followed by immunoblotting analysis with anti-V5 antibody to examine ubiquitination status of TAF7. **D,** HEK-293T cells that transfected with TAF7-WT or TAF7-K5R, K300R, 2KR mutants were subjected to CHX treatment for the specified time. Levels of SETD7 and TAF7 proteins were detected by western blotting analysis. **E,** HEK-293T cells were co-transfected with TAF7-WT+NC, TAF7-MUT+NC, TAF7-WT+siSETD7-1 or TAF7-MUT+siSETD7-1 and then treated with MG132 for 10 h. Co-IP assay with anti-His antibody followed by immunoblotting using anti-V5 antibody to detect ubiquitination levels of TAF7.

To clarify the function of SETD7-mediated TAF7 lysine methylation, we first examined the levels of TAF7 in SETD7 knockdown 786-O and CAKI-1 cells. Actually, TAF7 protein levels were sharply decreased after knockdown of SETD7 in ccRCC cell lines (Fig. S2A); however, the mRNA levels showed marginal changes (Fig. S2B). In order to further confirm whether SETD7 enhances TAF7 protein expression by blocking protein degradation, protein synthesis inhibitor cycloheximide (CHX) and proteasome inhibitor MG132 were used to detect TAF7 protein expression levels. We observed that SETD7 knockdown shortened the half-life of TAF7 when treated with CHX in ccRCC cells (Fig. 5A; Fig. S2C). Meanwhile, SETD7 knockdown significantly inhibited the expression of TAF7, and the addition of MG132 reversed the inhibitory effect of SETD7 knockdown on TAF7 protein (Fig. 5B). Additionally, Co-IP analyses showed that SETD7 knockdown can directly increase TAF7 ubiquitination and also indicated consistent results when HEK-293T cells were treated with PFI-2, the inhibitor of SETD7, suggesting that SETD may be responsible for TAF7 stability by decreasing its ubiquitination (Fig. 5C; Fig. S2D). Next, in order to explore whether the methylation of TAF7 K5 and K300 contributed to the stability of TAF7, WT, K5R, K300R as well as 2KR double mutants were respectively overexpressed in HEK-293T cells along with CHX treatment and we found the addition of CHX resulted in significantly shorter half-life of the three mutants than that of WT, indicating a reduction in TAF7 mutant protein levels (Fig. 5D; Fig. S2E). Additionally, Co-IP analyses showed that K5R, K300R and 2KR mutants displayed higher levels of ubiquitination than TAF7 WT (Fig. S2F). To further demonstrate that TAF7 K5 and K300 methylation inhibited ubiquitination, we added siRNAs or the inhibitor PFI-2 of SETD7 to HEK-293T cells overexpressing TAF7 WT or K5R, K300R, and 2KR mutants, respectively. The results showed the treatment with siRNAs led to TAF7 WT ubiquitination levels increased dramatically, however there were no significant changes in K5R, K300R and 2KR three mutants (Fig. 5E). Consistent results could be observed in HEK-293T cells with PFI-2 added (Fig. S2G). In short, our data provided a clear indication that SETD7-mediated methylation of TAF7 K5 and K300 prevents TAF7 ubiquitination, thereby promoting its stability.

### TAF7 serves as an oncogene to promote ccRCC progression

**Figure 6:**
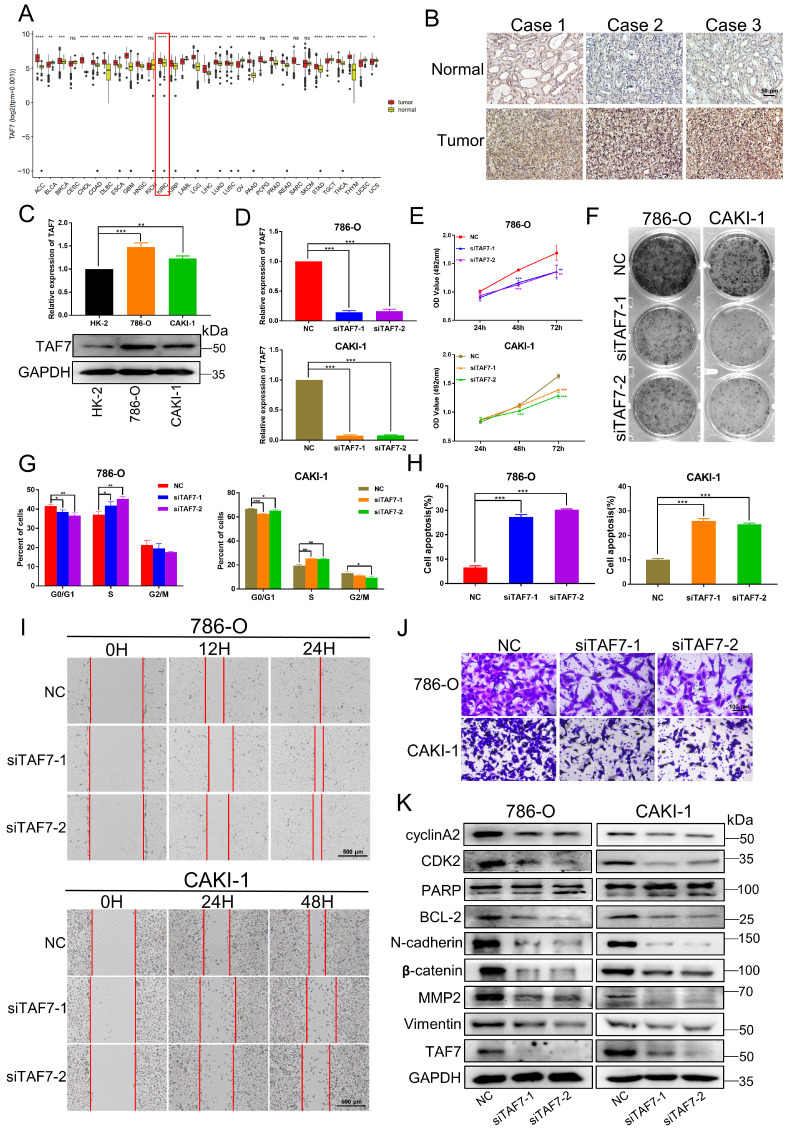
** Silencing of TAF7 inhibits the proliferation, migration and promoted apoptosis of ccRCC cells. A,** TCGA databases analyzed the expression of TAF7 in 31 Pan-cancer. **B,** TAF7 protein expression in ccRCC tissues vs normal tissues was confirmed by using immunohistochemistry assays. **C,** The mRNA and protein expressions of TAF7 in 786-O, CAKI-1 and HK-2 cells were detected by qRT-PCR and western blotting. **D,** The mRNA expression level of TAF7 in 786-O and CAKI-1 cells after si-TAF7 transfection, analyzed by qRT-PCR. **E, F,** The effect of silencing TAF7 on ccRCC cell proliferation was detected by MTT assay and colony formation assay. **G, H,** Flow cytometry statistical analysis was performed to detect the effects of silencing TAF7 on cell cycle progression and cell apoptosis in ccRCC cells. **I, J,** The effect of silencing TAF7 on ccRCC cell migration was detected by wound-healing assay and transwell assay. **K,** Expression changes of cell cycle, apoptosis and migration-related molecules in ccRCC cells after silencing TAF7, analyzed by western blotting. *p < 0.05, **p < 0.01, ***p < 0.001.

Next, we studied the biological function of TAF7 in ccRCC cells. Bioinformatics analysis showed TAF7 was significantly upregulated in a variety of tumors, including KIRC based on TCGA datasets (Fig. 6A). IHC staining confirmed that the expression of TAF7 protein in ccRCC tissues were remarkably higher than that of adjacent control tissues (Fig. 6B; Fig. S3A). As Fig. 6C shown, both mRNA and protein levels of TAF7 were also significantly higher expressed in ccRCC cells compared to normal control HK-2 cells. Then we constructed two siRNAs to silence TAF7, qRT-PCR and western blotting results indicated the knockdown efficiency of siRNAs could reduce the expression level of TAF7 by nearly 80% (Fig. 6D; Fig. S3B). Cell viability was determined by MTT assay in ccRCC cells and it was found that knockdown of TAF7 significantly inhibited cell proliferation (Fig. 6E). The results of clone formation assay also showed that inhibiting the expression of TAF7 would reduce the number and size of colonies formed (Fig. 6F; Fig. S3C). The results of flow cytometry on cell cycle and apoptosis showed that silencing TAF7 induced S-phase cell cycle arrest with a concomitant decrease in the number of G2/M phase cells and promoted both early and late cell apoptosis (Fig. 6G, H; Fig. S3D, E). Both wound-healing and transwell assays demonstrated that silencing TAF7 significantly suppressed the migration capacity of ccRCC cells compared to the control group (Fig. 6I, J; Fig. S3F, G). In addition, the potential molecular mechanism of TAF7 affecting ccRCC progression was detected by western blotting, as indicated by the results, the knockdown of TAF7 inhibited the levels of S-phase related proteins CDK2 and cyclin A2, thereby affecting cell cycle progression. At the same time, cleaved-PARP expression was elevated in TAF7-silenced cells, accompanied by a decrease in the expression of the anti-apoptotic protein BCL-2. Meanwhile, knockdown of TAF7 inhibited the levels of the migration-associated protein N-cadherin, β-catenin, MMP2 and Vimentin expression in ccRCC cells (Fig. 6K). The effects of siTAF7 on cell proliferation and migration and the molecular expression were basically in accordance with that of silencing SETD7 in ccRCC cells, which implied that TAF7 affected ccRCC progression as an oncogene.

### Restoration of TAF7 expression eliminates the effects of siSETD7 on ccRCC cells

**Figure 7:**
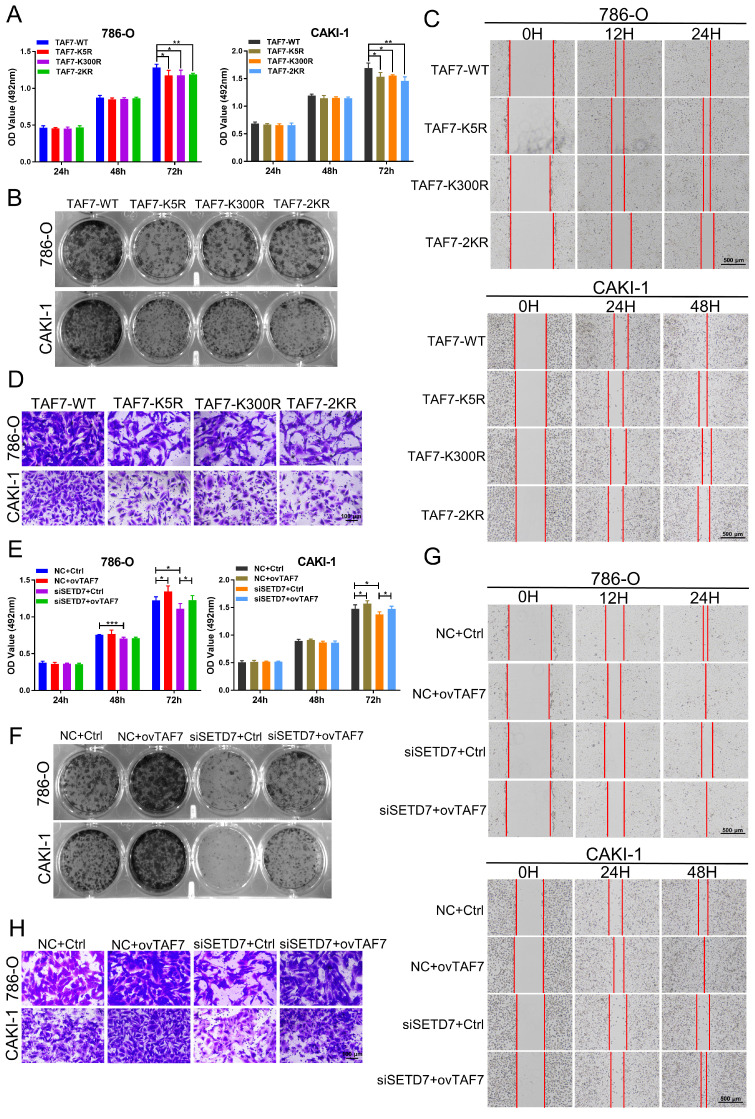
** Overexpression of TAF7 partially reverses the inhibition of ccRCC cell biological function caused by silencing SETD7. A, B,** MTT assays and colony formation assays were performed to determine the effect of TAF7-WT, TAF7-K5R, TAF7-K300R, or TAF7-2KR vectors treatment on cell viability in 786-O and CAKI-1 cells. **C, D,** Wound-healing assays and transwell analysis represented the migration capacity of ccRCC cells transfected with TAF7-WT, TAF7-K5R, TAF7-K300R or TAF7-2KR vectors, respectively. **E, F,** Cell activity levels of 786-O and CAKI-1 cells were examined by MTT and colony formation assays after co-transfection with NC+Ctrl, NC+ovTAF7, siSETD7+Ctrl, siSETD7+ovTAF7. **G, H,** The migration capacity was detected by wound-healing and transwell assays after co-transfected with NC+Ctrl, NC+ovTAF7, siSETD7+Ctrl, siSETD7+ovTAF7 in ccRCC cells. *p < 0.05, **p < 0.01, ***p < 0.001.

To further confirm the role of TAF7 in ccRCC cells, 786-O and CAKI-1 cells were transfected with TAF7 WT or K5R, K300R, and 2KR double mutant vectors, respectively. MTT and colony formation assays results showed TAF7 mutants, including K5R, K300R, and 2KR exhibited significantly weaker cell proliferation and colony formation capabilities compared to TAF7-WT (Fig. 7A, B; Fig. S4A). Both scratch assays and transwell assays found that the TAF7 mutants remarkably reduced the migration ability of ccRCC cells in comparison with TAF7-WT (Fig. 7C, D; Fig. S4B, C). Overall, mutant TAF7, including K5R, K300R, and 2KR presented relatively weaker cell progression than the TAF7-WT. In addition, we co-transfected 786-O and CAKI-1 cells with SETD7 siRNAs and TAF7 overexpressing plasmids to assess their potential counteracting effects on tumor cell proliferation and migration. As demonstrated by the results, knockdown of SETD7 inhibited, while overexpression of TAF7 promoted cell viability. More importantly, TAF7 overexpression partially reversed the inhibition of silencing SETD7 on ccRCC cell activity levels when co-transfected (Fig. 7E). In addition, the decrease in clonogenic ability of ccRCC cells caused by silencing SETD7 was also restored by co-transfection of TAF7 overexpression (Fig. 7F; Fig. S4D). The wound healing assay showed siSETD7 markedly suppressed the migration of ccRCC cells into the scratched area, while co-transfection with TAF7 overexpression significantly reversed the inhibitory effect of siSETD7 on cell migration (Fig. 7G; Fig. S4E). Consistent with the scratch results, the transwell assays also revealed the reduction in cell migration caused by knockdown SETD7 was also rescued by co-transfection with TAF7 overexpression (Fig. 7H; Fig. S4F).

To corroborate these results *in vivo*, we then generated shSETD7+TAF7-WT, shSETD7+TAF7-K5R as well as shSETD7+TAF7-K300R CAKI-1 cells with stable silencing of SETD7 while separately overexpressing TAF7 WT, K5R, and K300R for animal experiments. As shown in Fig. S4G and Fig. S4H, qRT-PCR and western blotting showed that both mRNA and protein of SETD7 were significantly downregulated, while both mRNA and protein of TAF7 were significantly upregulated in shSETD7+TAF7-WT, shSETD7+TAF7-K5R as well as shSETD7+TAF7-K300R cells compared to LV-Control cells. We subcutaneously injected shSETD7+TAF7-WT, shSETD7+TAF7-K5R and shSETD7+TAF7-K300R CAKI-1 cells into 5-week-old male BALB/C nude mice to evaluate tumor progression. At the 31th day after injection, the tumor formed of shSETD7+TAF7-K5R group or shSETD7+TAF7-K300R group were significantly smaller than shSETD7+TAF7-WT group (Fig. S4I, J). The tumor growth curve was plotted using tumor volume measured during tumor formation, and the results indicated that tumors in shSETD7+TAF7-K5R or shSETD7+TAF7-K300R infection group grew slower than in shSETD7+TAF7-WT group (Fig. S4K). As shown in Fig. S4L and S4M, the weight and volume of the excised solid tumors of shSETD7+TAF7-K5R or shSETD7+TAF7-K300R group were significantly smaller and lighter compared with shSETD7+TAF7-WT group. Lastly, qRT-PCR and western blotting of tumor tissues were performed to corroborate the relative mRNA and protein expression levels of CCNA2 in the three experimental groups. The results suggested that CCNA2 mRNA and protein levels in the shSETD7+TAF7-K5R or shSETD7+TAF7-K300R group were significantly down-regulated compared to the shSETD7+TAF7-WT group (Fig. S4N, O).

### Lysine methylation of TAF7 by SETD7 is crucial for CCNA2 gene activation

**Figure 8:**
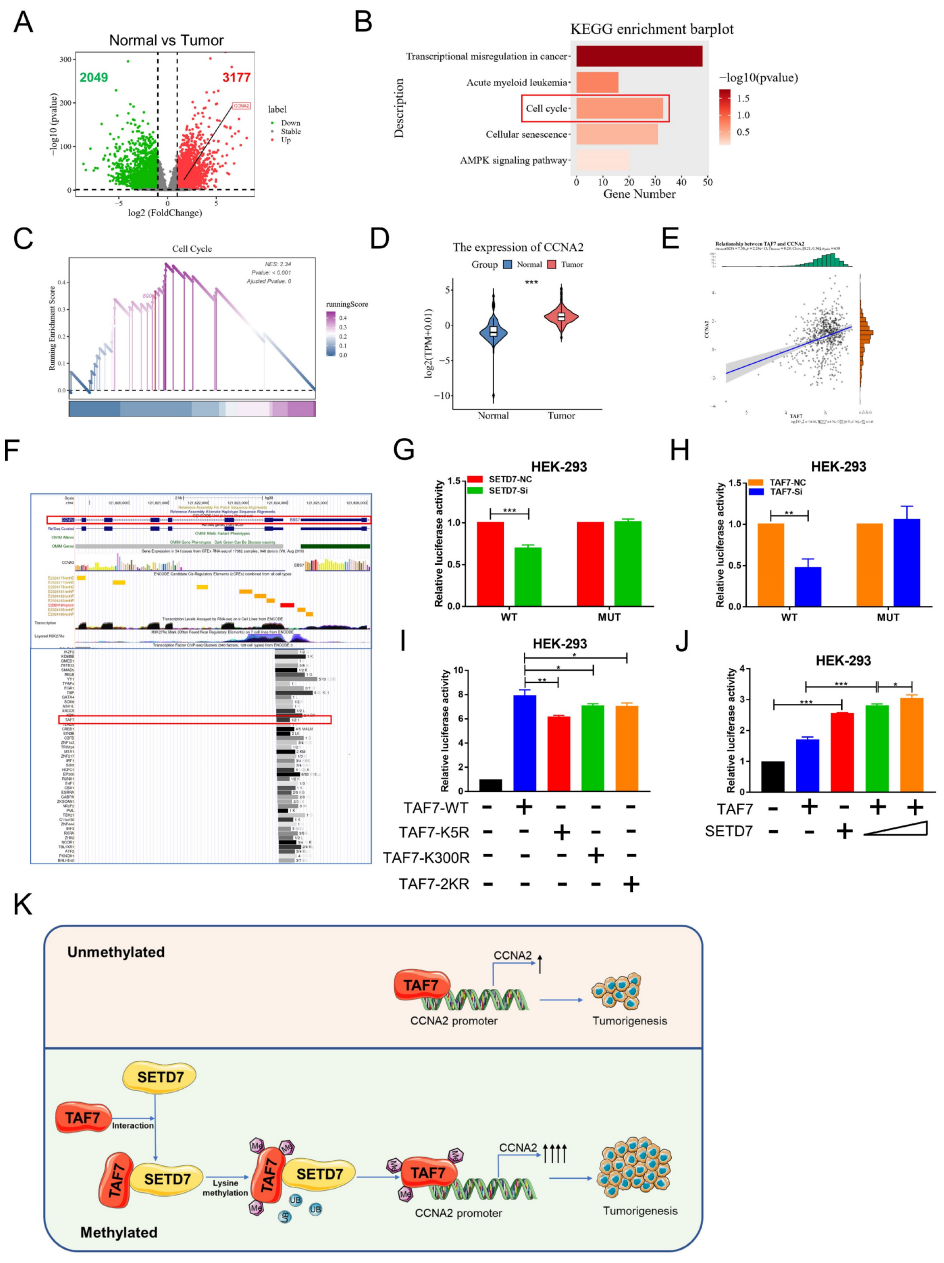
** TAF7 methylation enhances CCNA2 transcriptional and oncogenic activity. A,** Volcano plots for differentially expressed genes (DEGs) of ccRCC tumor versus normal kidney tissue samples. Red dots, significantly upregulated genes. Green dots, significantly downregulated genes. Grey dots, nondifferentially expressed genes. **B,** KEGG enrichment analysis for DEGs. **C,** GSEA snapshots of KEGG pathway enrichment analysis: Cell cycle. **D,** The expression of CCNA2 in ccRCC tissues and adjacent noncancerous kidney tissues in TCGA database. **E,** Scatterplot showed the correlation analysis between TAF7 and CCNA2 expression by Pearson's r. **F,** UCSC database analysis showed that TAF7 could bind to the promoter region of CCNA2 to regulate its transcription. **G,** Luciferase reporter assay were performed in HEK-293 cells to explore the effect on co-transfection with NC+pGL3-CCNA2-luc-WT, siSETD7+pGL3-CCNA2-luc-WT, NC+ pGL3-CCNA2-luc-MUT and siSETD7+pGL3-CCNA2-luc-MUT. Renilla luciferase served as the internal control. **H,** HEK-293 cells were co-transfected with NC+pGL3-CCNA2-luc-WT, siTAF7+pGL3-CCNA2-luc-WT, NC+pGL3-CCNA2-luc-MUT and siTAF7+pGL3-CCNA2-luc-MUT. Luciferase activity was determined at 48 h post-transfection. **I,** Luciferase assays were performed in HEK-293 cells after co-transfection with TAF7-WT or TAF7-MUT and pGL3-CCNA2-luc vector. Introduction of TAF7 mutants, including TAF7-K5R, TAF7-K300R and TAF7-2KR attenuated the transcriptional activation of the CCNA2 luciferase reporter compared with TAF7-WT. **J,** Luciferase activity in HEK-293 cells was measured 48 h after transfection with increasing amounts of SETD7. Introduction of SETD7 increased the transcriptional activation with CCNA2 in a dose-dependent manner. **K,** Proposed model for the regulation of ccRCC progression by the SETD7-TAF7-CCNA2 axis. SETD7 methylates TAF7 at K5 and K300, enhancing its binding to TAF7 and protecting TAF7 from ubiquitination degradation. The stabilization of TAF7 stimulates transcriptional expression activation of the target gene CCNA2, which consequently promotes the progression of ccRCC tumors.

The mechanism of transcription factors in tumor progression has been the focus of our research. Next, in order to explore downstream regulators of TAF7, we conducted a series of bioinformatics analyses. We first analyzed differentially expressed genes (DEGs) between ccRCC tumor versus normal kidney tissues according to the criteria of |log2FC| > 1 and *p* < 0.05. The results were visualized in volcano plots and we found 3177 genes were up-regulated, including CCNA2, and 2049 were down-regulated (Fig. 8A). Moreover, the KEGG analysis of these DEGs were enriched in 5 pathways (related to cancers), including transcriptional misregulation in cancer, Acute myeloid leukemia, cell cycle, cellular senescence and AMPK signaling pathway (Fig. 8B) and GSEA analysis showed that the selected gene sets were highly expressed in the pathway of cell cycle (Fig. 8C). Furthermore, CCNA2 presented a significant high expression trend in ccRCC tumor than in normal tissues (Figure 8D) and Pearson's r showed that TAF7 expression was positively correlated with CCNA2 expression (Figure 8E). UCSC database displayed that TAF7 could bind to the promoter region of CCNA2 to regulate its transcription (Figure 8F). Therefore, we hypothesized a potential binding between the transcription factor TAF7 and the CCNA2 promoter region. Subsequently, luciferase assays were performed to determine whether SETD7 or TAF7 can affect the activity of the target gene CCNA2. The wild-type (WT) or mutant (MUT) sequences of the CCNA2 promoter region were subcloned to upstream of the luciferase gene in the pGL3 reporter plasmid and co-transfected with siSETD7 or siTAF7 into HEK-293 cells and luciferase activity was measured 48 h after transfection. It was found that knockdown SETD7 or TAF7 significantly decreased the luciferase activity of WT vector compared with the NC groups, whereas MUT showed a negligible change upon the addition of siSETD7 or siTAF7, indicating the binding effect of TAF7 on the CCNA2 promoter region and positively regulate its transcription (Fig. 8G, H). To further investigate whether the TAF7-mediated regulation of downstream CCNA2 target genes was affected by TAF7 lysine methylation, luciferase assays continued to be performed utilizing TAF7-WT, K5R, K300R and 2KR mutants. Among the groups transfected first with the pGL3-CCNA2-luc plasmid and then transfected with TAF7-WT, TAF7-K5R, TAF7-K300R, or TAF7-2KR plasmids, respectively. The results suggested that luciferase activity was evidently reduced in the mutant groups, including K5R, K300R and double mutant 2KR compared with the TAF7-WT group, indicating that lysine methylation at the K5 and K300 sites of TAF7 enhance the transcriptional activity of TAF7 on CCNA2 gene (Fig. 8I). Next, to assess the importance of SETD7 for TAF7 methylation, we co-transfected SETD7 overexpression plasmids with TAF7-WT in HEK-293 cells and detected luciferase activity. Consistent with previous results that SETD7 could specifically enhance TAF7 methylation, SETD7 overexpression further activated target CCNA2 promoter activity, and SETD7 exerts a facilitating effect in a dose-dependent manner (Fig. 8J).

Furthermore, we explored the biological function of CCNA2 in ccRCC cells. As shown in Figure S5A, both mRNA and protein expression levels of CCNA2 were significantly higher in ccRCC cells compared with normal control HK-2 cells. Then we constructed two siRNAs to silence CCNA2, qRT-PCR and western blotting were performed to detect the knock-down efficiency and the results presented siRNAs could reduce the expression level of CCNA2 by nearly 90% (Fig. S5B). MTT assay showed that the cell proliferation was significantly inhibited by siCCNA2 compared with siNC transfection (Fig. S5C). In addition, the number and size of colonies formed were significantly reduced when CCNA2 was knocked down in 786-O and CAKI-1 cells (Fig. S5D).

The results of flow cytometry on cell cycle elucidated that silencing CCNA2 induced S-phase cell cycle arrest with a concomitant decrease in the number of G0/G1 phase cells (Fig. S5E). Meanwhile, we performed apoptosis analysis using flow cytometry and the results showed that silencing CCNA2 was found to significantly induce the ccRCC cell apoptosis (Fig. S5F), which was in line with the effects of TAF7 in ccRCC cells. In addition, the potential molecular mechanism of CCNA2 affecting ccRCC progression was detected by western blotting, as illustrated in Fig. S5G, the knockdown of CCNA2 inhibited the levels of S-phase related proteins CDK2, thereby affecting cell cycle progression. Besides, cleaved-PARP expression was elevated in CCNA2-silenced cells, accompanied by a reduction in the expression of the anti-apoptotic protein BCL-2.

Concurrently, to explore the effect of CCNA2 on the migration ability of ccRCC cells, wound-healing and transwell assays were performed. Consistent with TAF7, silencing of CCNA2 led to inhibition of cell migration. The wound healing rate of cells after silencing CCNA2 was significantly slower than that of the control group (Fig. S6A) and transwell assays also corroborated a hampered effect of silencing CCNA2 on cell migration capacity of ccRCC cells (Fig. S6B). Further exploration of the molecular mechanism by which CCNA2 modulates ccRCC progression revealed that the protein expression levels of N-cadherin, β-catenin and Vimentin were decreased by siCCNA2 (Fig. S6C).

In short, our results suggested that SETD7-dependent methylation of TAF7 triggers an increase in downstream target CCNA2 gene activation. Collectively, we could conclude that SETD7 stabilizes the TAF7 protein by methylating K5 and K300 residues of TAF7, enhancing its binding to TAF7 and protecting TAF7 from ubiquitination degradation. It further stimulated the TAF7-mediated transcriptional expression activation of the target gene CCNA2, thereby promoting the progression of ccRCC tumors (Fig. 8K).

## Discussion

Tumorigenesis is a multi-genetic, multi-stage, multi-step complex process involving the interaction between different pathways of different genes 27. Genetic imbalance in cancer cells and the research on its related molecular mechanisms has drawn more and more attention in recent years. Exploring the role of unknown genes in cancer and understanding their regulatory mechanisms is key to developing more effective biomarkers for the diagnosis and prognosis in cancer patients undergoing chemotherapies. According to previous reports and the Human Protein Atlas project, SETD7 was expressed differently in distinct types of tissues and cells. As a result, SET7 has two sides, such as acting as a tumor suppressor in lung cancer and yet as a tumor promoter in bladder cancer 28, 29. This depends on the type of cancer and its interacting partners. However, research on the functional role and molecular mechanisms of SETD7 in ccRCC is lacking. In this study, we showed that SETD7 was significantly highly expressed in ccRCC tissues and cell lines, which is highly consistent with the bioinformatics analysis results in pan-cancer. In addition, a series of cell function experiments showed that silencing the expression of SETD7 could inhibit the proliferation and migration of ccRCC cells *in vitro* and *in vivo*, implying that it may participate in the progression of ccRCC as an oncogene.

As a well-known methyltransferase, in addition to the modification and regulation of histones and chromatin, more importantly, SETD7 can also methylate many site-specific non-histone substrates, including numerous transcription factors 15, 16, 18. Transcription-related proteins are modified to alter their stability or regulate the activity of transcription factors and to recruit cofactors to gene promoter regions to regulate gene transcriptional activation. For example, in Hela cells, SETD7 has been found to monomethylate Yin Yang 1 (YY1) at K173 and K411 sites, and methylated YY1 has been shown to be involved in YY1-regulated gene transcription and cell proliferation. Conversely, substituting the K173 or K411 sites with arginine (K173R or K411R) weakens the binding activity of YY1 to DNA and completely eliminates the role of YY1 in promoting cell proliferation, demonstrating that SETD7-mediated YY1 methylation is involved in tumorigenesis 30. In non-small cell lung cancer (NSCLC), SETD7 can specifically methylate the Gli3 at K436 and K595 sites, increasing the stability and DNA binding ability of Gli3, thereby enhancing the activation of Shh signaling. In addition, functional experiments showed that SETD7-mediated methylation modification of Gli3 contributes to tumor growth and metastasis in NSCLC both *in vitro* and *in vivo* 31. A recent study revealed that the K162 site of PD-L1 can be methylated by SETD7 and demethylated by LSD2. K162 methylation of PD-L1 regulates PD-1/PD-L1 interaction, significantly enhances the inhibition of T cell activity controlling cancer immune surveillance 32, and another study reported that SETD7 primarily acts as a transcriptional inhibitor in prostate cancer by methylating the K270 site of FOXA1, which disrupts FOXA1-mediated transcription 33. Furthermore, SETD7 can specifically methylate the TBP-associated factor TAF10 at K189 site, which increases the stable recruitment of RNA pol II into promoters and stimulates transcription of target genes 34. Therefore, it is urgent and necessary to explore the potential novel substrates and methylation sites of SETD7, which is helpful to clarify the molecular mechanism of tumor progression and develop new therapeutic targets.

As a component of the general transcription factor complex TFIID, which coordinates transcription initiation and translation, TAF7 is involved in processes such as proliferation, cell cycle, metabolism, cytoskeleton organization, stress response and tumorigenicity 23, 35. As is reported in some studies, TAF7 is essential for embryonic development. Germline destruction of the TAF7 gene is embryologically fatal after 3.5 to 5.5 days. Mouse embryonic fibroblasts with TAF7 deletion halt transcription globally and stop proliferation, hinting at the importance of TAF7 for the survival and proliferation of embryonic cells 36. In addition, some studies noted that TAF7 was frequently overexpressed in various cancers through bioinformatics analysis and experimental verification including breast cancer 24, glioblastoma 25, lung adenocarcinoma 37 and colorectal cancer 38. Consistent with previous reports, our results indicated that TAF7 silencing inhibited ccRCC progression through reducing cell activity, increasing apoptosis, arresting S into G2/M cell cycle and diminishing migration, which substantiated the oncogenic capacity of TAF7 in ccRCC. However, little is known about the post-translational modifications of TAF7 protein, which may affect the stability, subcellular localization, activity and interactions of TAF7. Based on our findings, we showed that SETD7 and TAF7 were co-expressed in ccRCC cells. Further, by immunofluorescence and Co-IP analysis, we found that SETD7 and TAF7 were colocalized on the nucleus. More importantly, silencing of SETD7 inhibited the expression of the oncogene TAF7 in ccRCC cells, implying that SETD7 may play a role upstream of TAF7. Although a previous study tentatively proposed that SETD7 can methylate the TAF7 at K5 site 26. But importantly, in addition to the K5 site, we also discovered another novel site named K300, and conducted a series of verifications using COIP assays. These data indicated that SETD7 interacts with TAF7, which can serve as a novel methylated substrate for SETD7. However, whether methylation of TAF7 protein by SETD7 affects its ubiquitination and thus the stability remain unclear and still worth further exploration.

In eukaryotes, ubiquitination regulates intracellular signaling networks by triggering proteasome substrate degradation, altering substrate activity or mediating changes in proteins interacting with substrates 39. Ubiquitination is widely involved in the regulation of almost all life activities such as immune response regulation 40, DNA damage repair 41, cell cycle regulation 42, epigenetic regulation 43, cell proliferation 44, apoptosis 45 and protein degradation 46. To evaluate the hypothesis that SETD7 promotes TAF7 protein expression and stability by facilitating TAF7 protein methylation, we investigated the ability of SETD7 to prevent TAF7 protein degradation via ubiquitination. As our results showed, treatment with CHX, a potent inhibitor of protein synthesis, resulted in increasing TAF7 ubiquitination in SETD7 knockdown cells. In addition, treatment with the proteasome inhibitor MG132 significantly restored SETD7 knockdown-induced TAF7 protein reduction. Next, we specifically explored whether the stability of TAF7 protein altered after methylation of K5 and K300 sites. As results revealed, SETD7 could methylate TAF7 at K5 and K300 sites, while methylation enhances the stability of TAF7 protein, protecting it from ubiquitination degradation. Besides, a series of co-transfection rescue experiments confirmed that TAF7 overexpression could reverse the inhibition of silencing SETD7 on the proliferation and migration of ccRCC cells, which corroborated the regulatory relationship of SETD7 on TAF7 at the functional level. At the same time, qRT-PCR and western blotting were conducted to detect mRNA and protein expression levels of SETD7 after silencing TAF7. The results revealed that SETD7 showed negligible changes at both RNA and protein levels following TAF7 knockdown in ccRCC cells. Therefore, we speculated that TAF7 has a weak regulatory effect on SETD7, and there may be no feedback loop between the two, but a one-way regulation of TAF7 stability by SETD7.

Cyclin A2, also known as CCNA2, belongs to the cyclin family and regulates cell cycle progression by interacting with CDK kinases 47. Increased CCNA2 expression has been observed in many types of cancer, including colorectal cancer 48, non-small cell lung cancer 49, triple negative breast cancer 50 and ovarian carcinoma 51. Bioinformatics analysis prompted that TAF7 may have a potential regulatory effect on CCNA2 25. In our study, silencing TAF7 arrested the cell cycle from the S to G2/M phase and significantly reduced the luciferase activity of WT vector, hinting the binding effect of TAF7 on the S phase-associated protein CCNA2 promoter region and positively regulate its transcription. Overall, our study highlights novel regulatory mechanisms for SETD7 methylated TAF7 at K5 and K300 sites to enhance its protein expression and stability. At the same time, we uncover the novel critical function of the SETD7-TAF7-CCNA2 axis in ccRCC, potentially providing a basis for developing effective therapeutic strategies by targeting members of this axis.

## Conclusion

In conclusion, the present study has depicted a series of molecular events in which SETD7 methylates and stabilizes TAF7, providing a direct link between lysine methylation and deubiquitination, leading to the transcriptional activation of CCNA2 genes. Consequently, these reactions result in the promotion of proliferation and migration of ccRCC cells, and facilitate tumor growth and metastasis *in vivo*. These findings may provide promising therapeutic strategies for ccRCC cancer.

## Supplementary Material

Supplementary figures and tables.

## Abbreviations

SETD7: SET domain containing 7
RCC: Renal cell carcinoma
ccRCC: Clear cell renal cell carcinoma
TAF7: TATA-box binding protein associated factor 7
IF: Immunofluorescence
CO-IP: Co-immunoprecipitation
IHC: Immunohistochemistry
qRT-PCR: Quantitative real-time PCR
WT: Wild-type
MUT: Mutant
TCGA: The Cancer Genome Atlas
DEGs: Differentially expressed genes
