# Supplementary material for: SETD7 Promotes Cell Proliferation and Migration via Methylation-mediated TAF7 in Clear Cell Renal Cell Carcinoma
Source: Int J Biol Sci. 2024 May 19;20(8):3008–27. doi: 10.7150/ijbs.93201 (PMC11186372; doi:10.7150/ijbs.93201)
Supplement: Supplementary file 1 — Supplementary figures and tables. [file ijbsv20p3008s1.pdf]

# Supplementary Material

## Supplementary table:

**Table S1. The URL of the bioinformatics database and the version information of the drawing package in this work.**

| Name | Description |
|---|---|
| TCGA | https://github.com/openbiox/XenaShiny |
| String | https://cn.string-db.org/ |
| GEPIA | http://gepia.cancer-pku.cn/ |
| UCSC | https://genome.ucsc.edu/ |
| R package of ggplot 2 | version:3.4.4 |
| R package clusterProfiler | version: 4.7.1.003 |

**Table S2. Primers and oligonucleotides used in this work.**

| Name | Sequence (5’-3’) |
|---|---|
| siNC-S | UUCUCCGAACGUGUCACGUTT |
| siNC-A | ACGUGACACGUUCGGAGAATT |
| siSETD7-1S | GCCUUGUAGGAGAAGUAAATT |
| siSETD7-1A | UUUACUUCUCCUACAAGGCTT |
| siSETD7-2S | CCCCUGUUUUUUUAGUUUATT |
| siSETD7-2A | UAAACUAAAAAAACAGGGGTT |
| siTAF7-1S | GUGGAAAAUCUGGCUCUCATT |
| siTAF7-1A | UGAGAGCCAGAUUUUCCACTT |
| siTAF7-2S | GGACAAAGAGAAAAAGUUUTT |
| siTAF7-2A | AAACUUUUUCUCUUUGUCCTT |
| siCCNA2-1S | GGAUCUUCCUGUAAAUGAUTT |
| siCCNA2-1A | AUCAUUUACAGGAAGAUCCTT |
| siCCNA2-2S | GAAGCCAGCUGAAUCUCAATT |
| siCCNA2-2A | UUGAGAUUCAGCUGGCUUCTT |
| SETD7-F | CGAATTACACACCAAGAGGTT |
| SETD7-R | TAGGCAACGGTGAGCTCTTC |
| TAF7-F | AGCTTCGGGAGATATTCA |
| TAF7-R | TTTCCTGGTGCTGTTCAT |
| CCNA2-F | CTCTACACAGTCACGGGACAAAG |
| CCNA2-R | CTGTGGTGCTTTGAGGTAGGTC |
| GAPDH-F | GGAGCGAGATCCCTCCAAAAT |
| GAPDH-R | GGCTGTTGTCATACTTCTCATGG |

**Table S3. Antibodies and inhibitors information in this work.**

| Name | Company | Catalog No. |
|---|---|---|
| SETD7 | Abcam | ab124708 |
| SETD7 | Proteintech | 24840-1-AP |
| GAPDH | Abways | AB0038 |
| cyclinA2 | Proteintech | 18202-1-AP |
| CDK2 | Proteintech | 10122-1-AP |
| PARP | Proteintech | 13371-1-AP |
| Caspase 9 | Proteintech | 10380-1-AP |
| BCL-2 | Proteintech | 12789-1-AP |
| BAX | Proteintech | 50599-2-Ig |
| N-cadherin | Proteintech | 22018-1-AP |
| β-catenin | Proteintech | 51067-2-AP |
| MMP2 | Proteintech | 10373-2-AP |
| Vimentin | Proteintech | 10366-1-AP |
| TAF7 | Proteintech | 13506-1-AP |
| Flag | Proteintech | 20543-1-AP |
| His | Proteintech | 66005-1-Ig |
| Rabbit IgG | Proteintech | B900610 |
| Mouse IgG | Proteintech | B900620 |
| Methylated Lysine | PTMBIO | PTM-602 |
| V5 | Proteintech | 14440-1-AP |
| Goat Anti-Rabbit IgG (H+L) HRP | Abways | AB0101 |
| Goat Anti-Mouse IgG (H+L) HRP | Abways | AB0102 |
| Rabbit Anti-Mouse IgG Kappa Light Chain | Proteintech | SA00001-19 |
| Mouse Anti-Rabbit IgG, Light Chain | Proteintech | SA00001-7L |
| Cycloheximide (CHX) | MCE | HY-12320 |
| MG132 | MCE | HY-13259 |
| PFI-2 | MCE | HY-18627A |

## Supplementary figure:

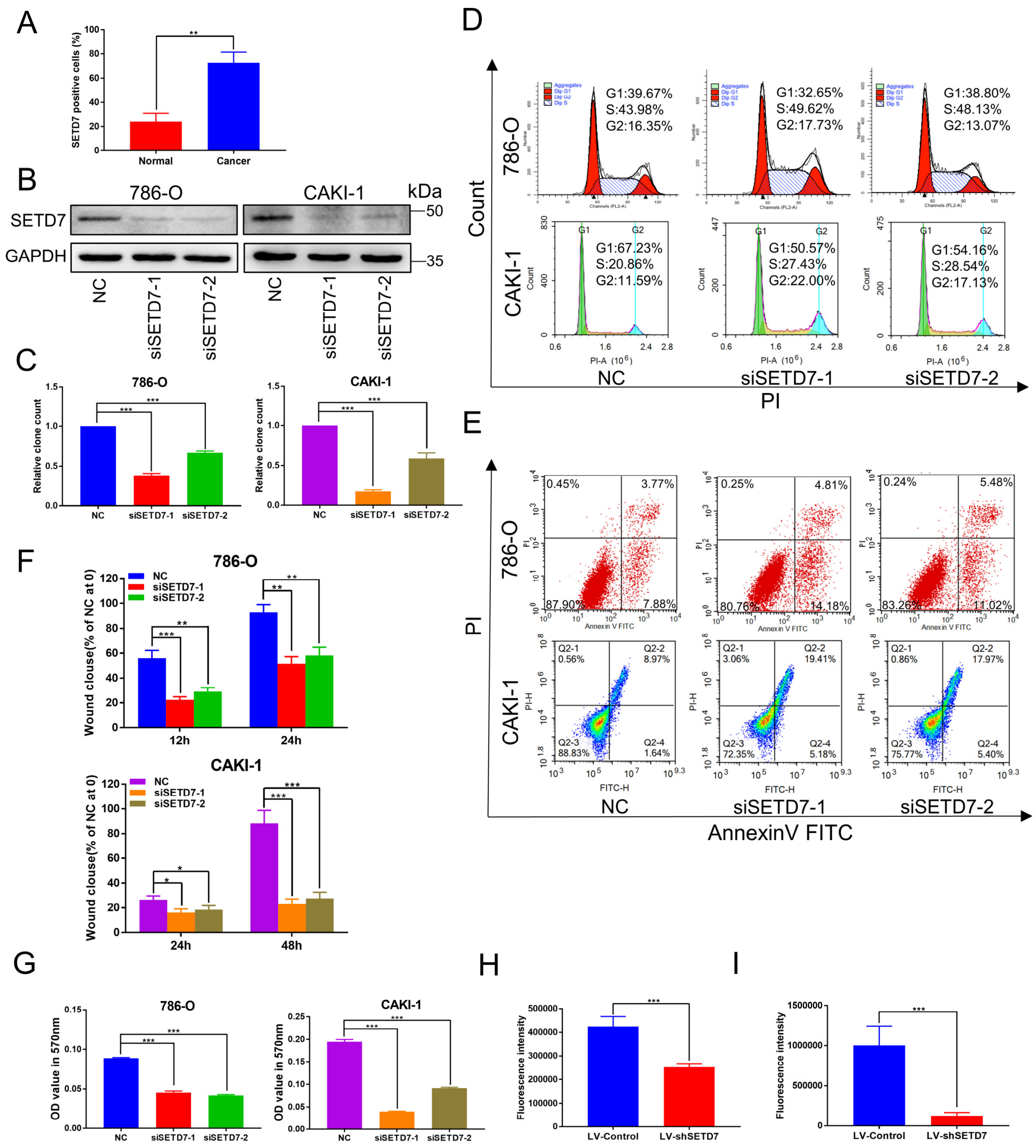

**Figure S1. SETD7 silencing suppresses ccRCC cell proliferation and migration in vitro and in vivo. A,** Quantification of immunohistochemical staining of SETD7 positive cells. **B,** Relative SETD7 protein expression level in 786-O and CAKI-1 cells after siSETD7 transfection, analyzed by western blotting. **C,** Quantification of colony formation assay in 786-O and CAKI-1 cells transfected with SETD7 siRNAs. **D,** Representative images of flow cytometry of cell cycle progression in 786-O and CAKI-1 cells after silencing SETD7. **E,** Representative images of flow cytometry of cell apoptosis in ccRCC cells after silencing SETD7. **F,** Quantification of wound-healing assay in 786-O and CAKI-1 cells transfected with SETD7 siRNAs. **G,** Quantitative analysis of the migration rates by solubilization of crystal violet and spectrophotometric reading at OD 570 nm. **H,** Quantitative analysis of fluorescence intensity in nude mice injected with LV-Control group and LV-shSETD7 group, respectively. **I,** Quantification of fluorescence intensity of resected organs. *$p < 0.05$, **$p < 0.01$, ***$p < 0.001$.

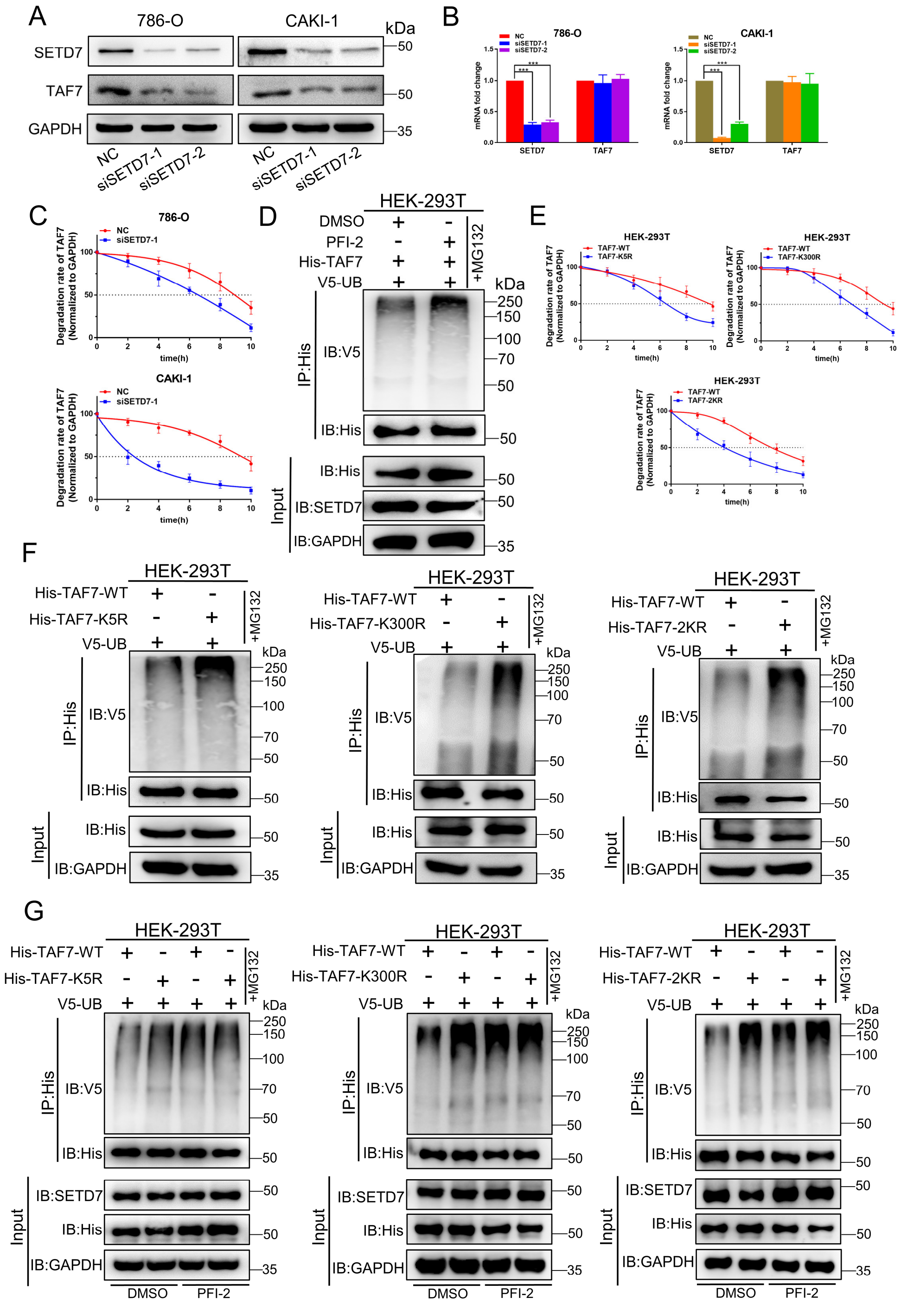


**Figure S2. TAF7 K5 and K300 methylation promote the stabilization of TAF7. A, B,** Protein and relative mRNA expression levels of TAF7 in 786-O and CAKI-1 cells transfected with NC or SETD7 siRNAs were assessed by western blotting (A) and qRT-PCR analysis (B). **C,** Quantification of TAF7 half-life changes in NC and SETD7 knockdown 786-O and CAKI-1 cells treated with CHX for the indicated time. **D,** HEK-293T cells were treated with or without PFI-2 for 48 h and MG132 for 10 h, and cell lysates were immunoprecipitated (IP) using anti-His antibody and subsequently analyzed by immunoblotting (IB). **E,** Quantification of TAF7 half-life changes in HEK-293T cells that transfected with TAF7-WT or TAF7-K5R, K300R, 2KR mutants and subjected to CHX treatment for the specified time. **F,** HEK-293T cells were transfected with TAF7-WT, TAF7-K5R, TAF7-K300R or TAF7-2KR vectors, respectively and then treated with MG132 for 10 h. Cell lysates were IP using anti-His antibody and then subjected to IB. **G,** HEK-293T cells expressing TAF7 WT, TAF7-K5R, TAF7-K300R or TAF7-2KR, respectively, were treated with or without PFI-2 for 48 h and MG132 for 10 h. Co-IP assay was performed using anti-His antibody, following by IB to assess the ubiquitination level of TAF7.

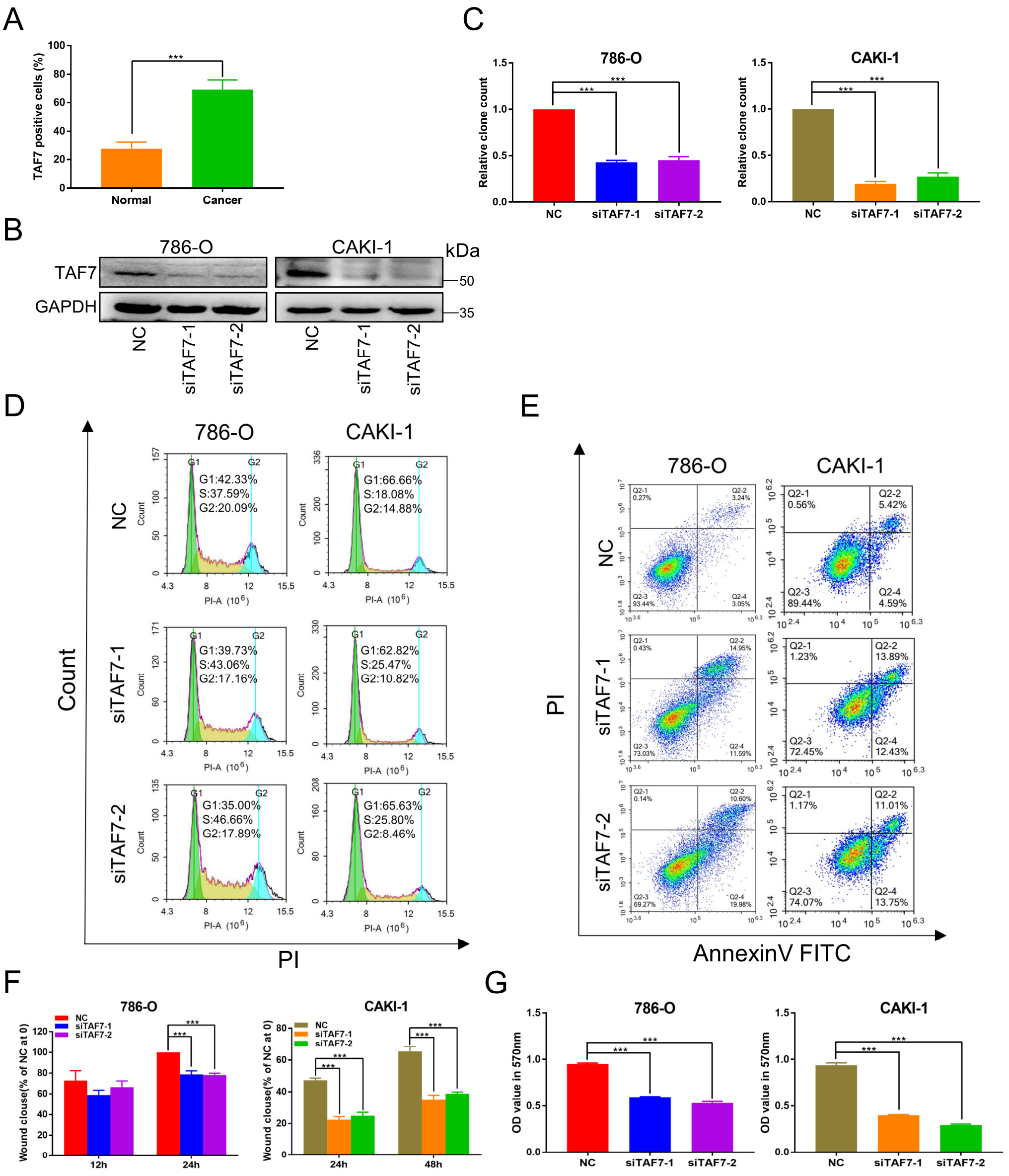


**Figure S3. TAF7 silencing inhibits the proliferation, migration and promotes apoptosis of ccRCC cells. A,** Quantitative analysis of immunohistochemical staining of TAF7 positive cells. **B,** Western blotting analysis of TAF7 in 786-O and CAKI-1 cells transfected with NC and TAF7 siRNAs. **C,** Quantification of colony formation assay in ccRCC cells transfected with TAF7 siRNAs. **D,** Representative images of flow cytometry of cell cycle in 786-O and CAKI-1 cells after silencing TAF7. **E,** Representative images of flow cytometry of cell apoptosis in ccRCC cells after knockdown TAF7. **F,** Quantitative analysis of wound-healing assay in 786-O and CAKI-1 cells after transfection with TAF7 siRNAs. **G,** Quantification of the migration rates by dissolving the crystal violet and spectrophotometric reading at OD 570 nm. *$p < 0.05$, **$p < 0.01$, ***$p < 0.001$.

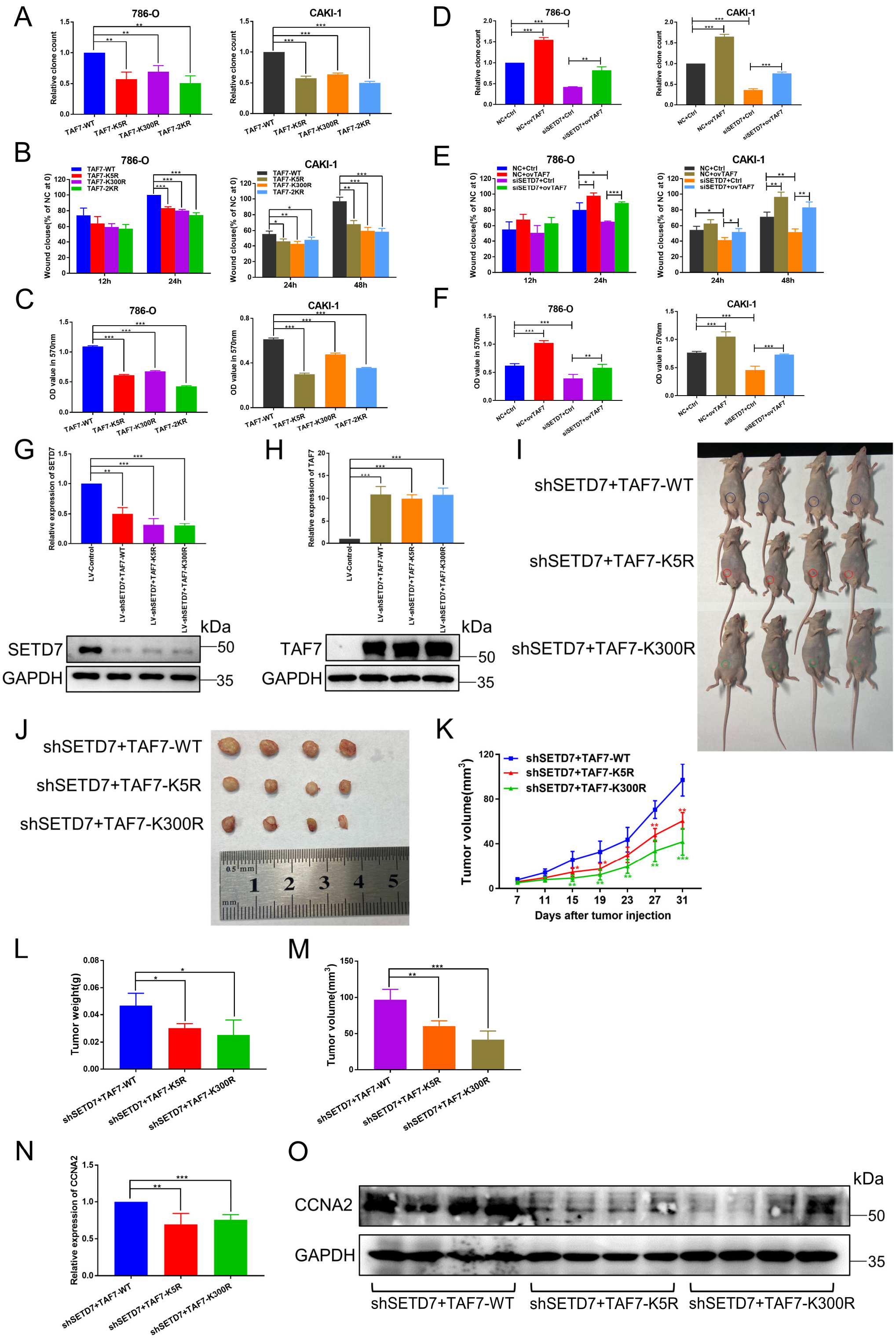


**Figure S4. The expression of TAF7 reverses the cell function changes induced by si-SETD7. A,** Quantitative analysis of colony formation assay in 786-O and CAKI-1 cells transfected with TAF7-WT, TAF7-K5R, TAF7-K300R or TAF7-2KR. **B,** Quantification of wound-healing assay in ccRCC cells after transfection with TAF7-WT or TAF7-mutant plasmids. **C,** Quantitative analysis of the migration rates by solubilization of crystal violet and spectrophotometric reading at OD 570 nm. **D,** Quantification of colony formation assay in 786-O and CAKI-1 cells after co-transfection with NC+Ctrl, NC+ovTAF7, siSETD7+Ctrl, siSETD7+ovTAF7. **E,** Quantification of wound-healing assay in ccRCC cells co-transfected with NC+Ctrl, NC+ovTAF7, siSETD7+Ctrl, siSETD7+ovTAF7. **F,** Quantitative analysis of the migration rates by dissolving the crystal violet and spectrophotometric

reading at OD 570 nm. **G:** The mRNA and protein expression of SETD7 in shSETD7+TAF7-WT, shSETD7+TAF7-K5R as well as shSETD7+TAF7-K300R cells were detected by qRT-PCR and western blotting. **H:** The mRNA and protein expression of TAF7 in shSETD7+TAF7-WT, shSETD7+TAF7-K5R and shSETD7+TAF7-K300R cells were detected by qRT-PCR and western blotting. **I:** Gross morphology of tumors injected with either shSETD7+TAF7-WT, shSETD7+TAF7-K5R or shSETD7+TAF7-K300R cells after 31 days (n = 4/group). **J:** Gross morphology of excised tumors from nude mice (n = 4/group). **K:** Growth curves of xenograft by volume every 4 days (from day 7 to day 31). **L:** Quantification of xenograft weight after tumor extraction on day 31. **M:** Quantification of xenograft volume after tumor excision on day 31. **N:** The mRNA expression levels of CCNA2 was analyzed by qRT-PCR in xenograft tumor tissues. **O:** The protein expression levels of CCNA2 was analyzed by western blotting in removed tumor tissues. $^{*}p < 0.05$, $^{**}p < 0.01$, $^{***}p < 0.001$.

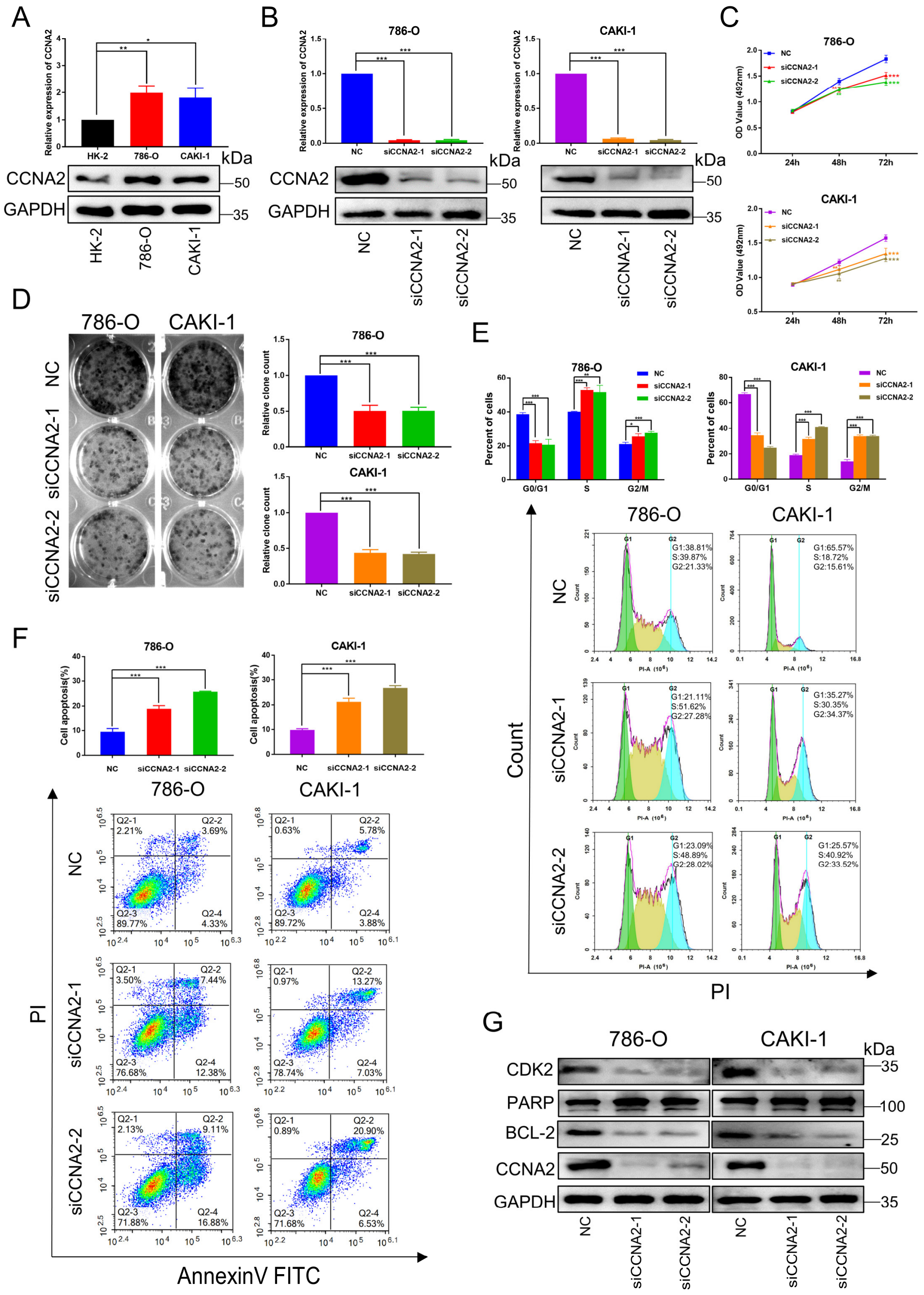


**Figure S5. CCNA2 knockdown inhibits proliferation of ccRCC cells. A,** CCNA2 mRNA and protein expression levels were detected in ccRCC cell lines (786-O, CAKI-1) and normal tubular epithelial cell line (HK-2), analyzed by qRT-PCR and western blotting. **B,** The mRNA and protein expression of CCNA2 were measured to confirm the knockdown efficiency of two siRNAs in ccRCC cells, analyzed by qRT-PCR and western blotting. **C,** The effects of CCNA2 on ccRCC cell proliferation were determined by MTT assay at 24, 48 and 72 h. **D,** Representative results of colony formation of ccRCC cells after silencing CCNA2. **E,** Flow cytometry was performed to detect the effects of silencing CCNA2 on cell cycle progression in ccRCC cells, and the percentages of G1, S, G2 phase of cell cycle were calculated. **F,** The effects of silencing CCNA2 on cell apoptosis was detected by Flow cytometry in ccRCC cells, and the percentage of cell apoptosis was calculated. **G,** Expression changes of S phase and apoptosis-related molecules in ccRCC cells after silencing CCNA2, detected by western blotting. *$p < 0.05$, **$p < 0.01$, ***$p < 0.001$.

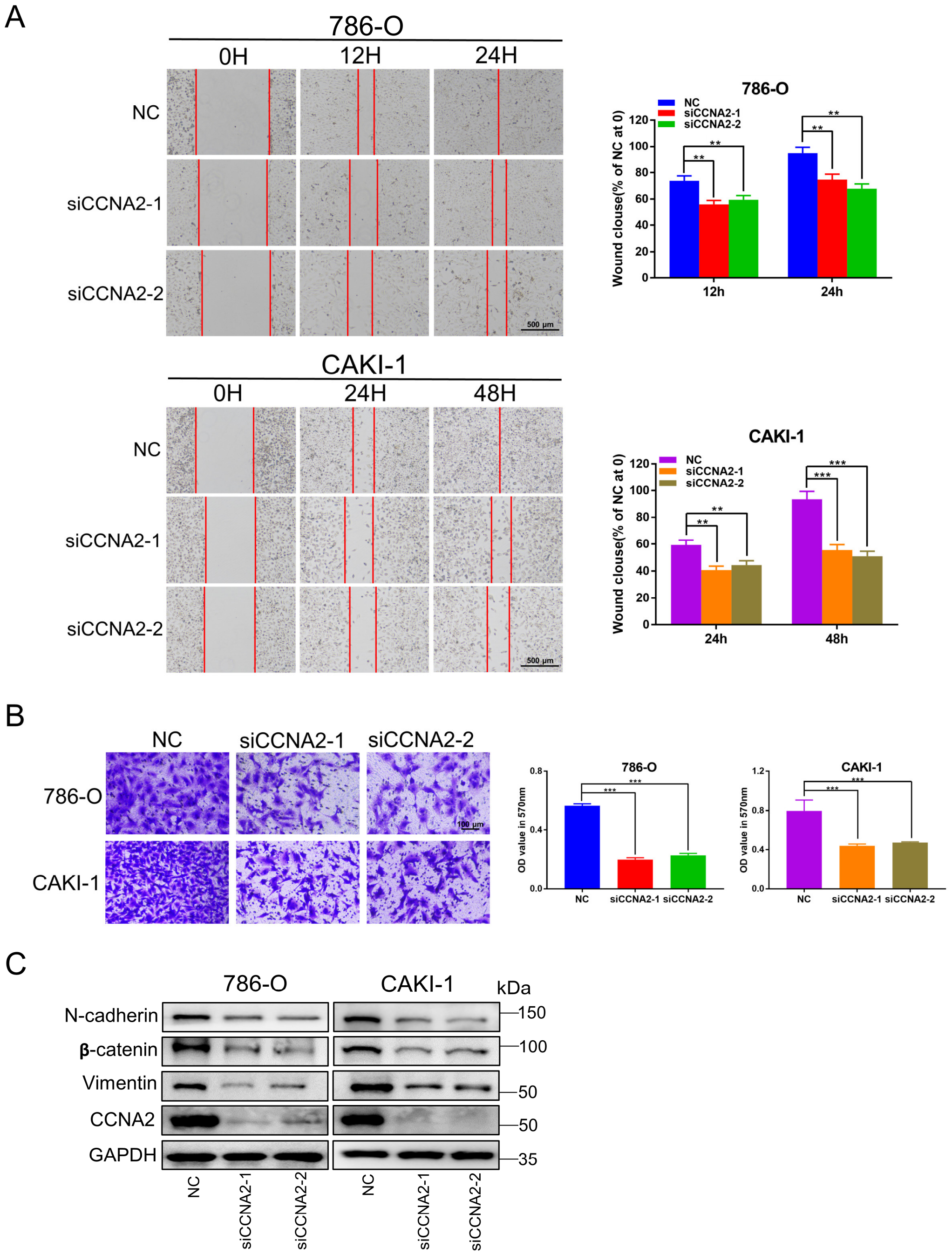


**Figure S6. CCNA2 knockdown inhibits migration of ccRCC cells. A,** Wound-healing assays were performed to detect the effects of silencing CCNA2 on cell migration in ccRCC cells. **B,** Transwell assay analysis of ccRCC cells after silencing CCNA2. **C,** Western blotting demonstrated that the expressions of N-cadherin, β-catenin and Vimentin were decreased following CCNA2 knockdown in both 786-O and CAKI-1 cells. *$p < 0.05$, **$p < 0.01$, ***$p < 0.001$.
